# Proteomic analysis of peach fruit mesocarp softening and chilling injury using difference gel electrophoresis (DIGE)

**DOI:** 10.1186/1471-2164-11-43

**Published:** 2010-01-18

**Authors:** Ricardo Nilo, Carlos Saffie, Kathryn Lilley, Ricardo Baeza-Yates, Verónica Cambiazo, Reinaldo Campos-Vargas, Mauricio González, Lee A Meisel, Julio Retamales, Herman Silva, Ariel Orellana

**Affiliations:** 1 Centro de Biotecnología Vegetal, Universidad Andrés Bello, Santiago, Chile; 2 Millennium Nucleus in Plant Cell Biotechnology (MN-PCB), Santiago, Chile; 3 Cambridge Centre for Proteomics, University of Cambridge, Cambridge, UK; 4 Dept. of Computer Science, Universidad de Chile, Santiago, Chile; 5 Laboratorio de Bioinformática y Expresión Génica, INTA-Universidad de Chile, Santiago, Chile; 6 Millennium Nucleus Center for Genomics of the Cell (CGC), Santiago, Chile; 7 Institute of Agricultural Research (INIA-La Platina), Santiago, Chile; 8 Facultad de Ciencias Agronómicas, Universidad de Chile, Santiago, Chile; 9 Plant Functional Genomics & Bioinformatics Lab, Universidad Andrés Bello, Santiago, Chile

## Abstract

**Background:**

Peach fruit undergoes a rapid softening process that involves a number of metabolic changes. Storing fruit at low temperatures has been widely used to extend its postharvest life. However, this leads to undesired changes, such as mealiness and browning, which affect the quality of the fruit. In this study, a 2-D DIGE approach was designed to screen for differentially accumulated proteins in peach fruit during normal softening as well as under conditions that led to fruit chilling injury.

**Results:**

The analysis allowed us to identify 43 spots -representing about 18% of the total number analyzed- that show statistically significant changes. Thirty-nine of the proteins could be identified by mass spectrometry. Some of the proteins that changed during postharvest had been related to peach fruit ripening and cold stress in the past. However, we identified other proteins that had not been linked to these processes. A graphical display of the relationship between the differentially accumulated proteins was obtained using pairwise average-linkage cluster analysis and principal component analysis. Proteins such as endopolygalacturonase, catalase, NADP-dependent isocitrate dehydrogenase, pectin methylesterase and dehydrins were found to be very important for distinguishing between healthy and chill injured fruit. A categorization of the differentially accumulated proteins was performed using Gene Ontology annotation. The results showed that the 'response to stress', 'cellular homeostasis', 'metabolism of carbohydrates' and 'amino acid metabolism' biological processes were affected the most during the postharvest.

**Conclusions:**

Using a comparative proteomic approach with 2-D DIGE allowed us to identify proteins that showed stage-specific changes in their accumulation pattern. Several proteins that are related to response to stress, cellular homeostasis, cellular component organization and carbohydrate metabolism were detected as being differentially accumulated. Finally, a significant proportion of the proteins identified had not been associated with softening, cold storage or chilling injury-altered fruit before; thus, comparative proteomics has proven to be a valuable tool for understanding fruit softening and postharvest.

## Background

Fruit softening is a complex process during which a large number of proteins interact in order to achieve the physiological condition that allows fruit to accomplish its final objective, seed dispersion [1-3]. Peaches (*Prunus persica *L. Bastch) from fresh eating melting flesh varieties are characterized as having a short shelf life due to the rapid loss of firmness at the end of the ripening process, the softening of the fruit mesocarp. Changes in the cell wall are particularly important for this phenomenon, especially the dismantling of its structure, the degradation of the polymers of which it is composed and the loss of turgor pressure in the fruit [4]. After softening, fruit is susceptible to physical injury and pathogen attack and can only be stored for a few days [5].

Cold storage has been used to increase the postharvest life of peach fruit; however, this procedure leads to undesirable changes in fruit quality. These symptoms are known as chilling injury and include mealiness or lack of juice, as well as browning, among others [6,7]. Mealiness has been associated with abnormal cell wall dismantling during cold storage and the subsequent ripening. However, the mechanisms that disrupt the normal fruit cell wall metabolism during this low temperature disorder are not yet clear [6]. The browning phenotype has been linked to interaction between phenols and polyphenol oxidase. These elements are usually found in different compartments within the cell, but the membrane permeability of deteriorated tissues may cause them to come into contact with one another [6]. Other important metabolic processes may be altered in cold injured fruit. For example, there is evidence that abnormal oxidative metabolism is triggered at low storage temperatures [8-11]. Despite these findings, little is known about the softening process and the molecular events that lead to the physiological disorder observed in cold stored fruit.

In order to increase our understanding of this physiological process, we engaged in a global analysis using a proteomic approach that should provide us with a more accurate representation of the cellular/physiological state of the fruit rather than profiling the expression of mRNAs [12,13]. Our aim was to identify proteins that could be differentially accumulated in soften and/or cold injured peaches. To this end, we utilized two-dimensional gel electrophoresis (2-D), a widely used technology that allows for the evaluation of many proteins. Several steps of the process have been optimized during recent years, giving this technique an excellent resolving power [14,15]. Furthermore, the introduction of 2-D difference gel electrophoresis (DIGE) by Ünlü et al. [16], allows the comparison of different protein samples within the same gel, strengthening the confidence of the 2-D quantitative analysis. Thus, a 2-D DIGE approach was designed to inspect for proteins that could be differentially accumulated in peach fruit during softening and under conditions that led to chilling injury altered fruit.

## Results

### Physiological response of peach fruit to different postharvest storage conditions

**Figure 1:**
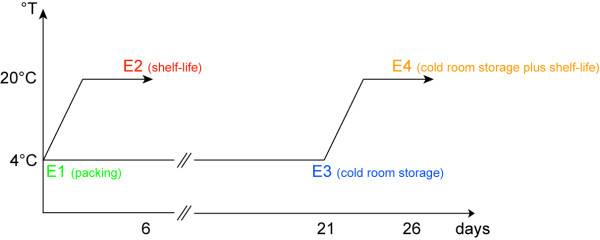
**Schematic representation of the postharvest conditions under evaluation in this study**. In order to acquire information about the normal softening process of *P. persica *fruit, two postharvest conditions were selected and assigned as E1 and E2. Stage E1 (represented by the green colour, day 0) was used to name fruit that went through packing whereas fruit that was allowed to ripen at 20°C, after packing, was named as E2 (represented by the red colour, day 6). The dataset acquired from these conditions was compared to the dataset from the postharvest conditions E3 and E4, which ultimately leads to the development of chilling injury in the fruit mesocarp. E3 (represented by the blue colour), was used to name fruit that was cold stored for 21 days and E4 (represented by the orange colour, day 26), corresponded to fruit that went through cold storage and then was allowed to ripen at 20°C.

The peaches utilized in this work correspond to those analyzed by Campos-Vargas et al [7]. In that study four postharvest stages were selected and each group named as: E1, firm fruit taken from packing; E2, fruit soften at 20°C; E3, fruit stored in a cold room at 4°C for three weeks; and E4, fruit stored at cold room and then kept at 20°C to allow for softening (Figure 1). Some of the physiological parameters measured by Campos-Vargas et al. [7] are summarized in Table 1, which also describes the degree of browning of the fruit at each stage. The results showed that fruit became nearly ten times less firm under normal softening conditions, dropping from 72.5 N in E1 to 7.9 N in E2. This change is characteristic of peach melting varieties. Fruit stored at 4°C for three weeks (E3) showed a significant decrease in firmness, reaching a value of 43.2 N. Fruit from the E4 stage reached a firmness value of 6.8 N, which is similar to normally softened fruit (Table 1, which also describes the degree of browning of the ). The fruit from the E4 stage showed symptoms of chilling injury such as lack of juice (Table 1) and browning (Table 1, additional file 1). Peaches that were softened after being stored in the cold (E4) produced more ethylene than fruit softened normally. As expected, respiration also increased from E1 to E2. Interestingly, fruit kept in the cold (E3) exhibited a high respiration rate that did not change when the fruit was allowed to soften (E4; Table 1).

**Table 1:** Fruits maturity and physiological parameters.

Sample	Firmness (N)	TSS (%)	Respiration Rate (mL CO_2 _kg-1 h-1)	Ethylene production (μL C_2_H_4 _kg-1 h-1 )	% juice	Browning degree^3^
Packing	72.5^a^	10.7^a^	28.8^a^	1.6^a^	-^2^	+
Packing + ripening	7.9^b^	10.6^a^	110.8^b^	4.7^a^	31.3^a^	++
Stored at 4°C	43.2^c^	11.1^a^	94.3^c^	1.0^a^	-^2^	+
Stored at 4°C + ripening	6.8^b^	-^1^	98.2^c^	18.1^b^	0.7^b^	+++/++++

TSS - Total soluble solids

^a,b,c ^Values (means) followed by different small letters are significantly different within the same column at P = 0.05

^1^: No sample available for measurements

^2 ^: Juice can not be measured in these samples due to the high firmnes they present

^3^: Please refer to additional file 1 for further details; + no browning; ++ no browning degree with a color change of the mesocarp; +++ medium browning degree; ++++ high browning degree

The data used in this table have been acquired from Campos-Vargas et al. Sci Hortic-Amsterdam 2006, 110(1):79-83

### 2-D map of peach mesocarp fruit developed using DIGE technology

**Figure 2:**
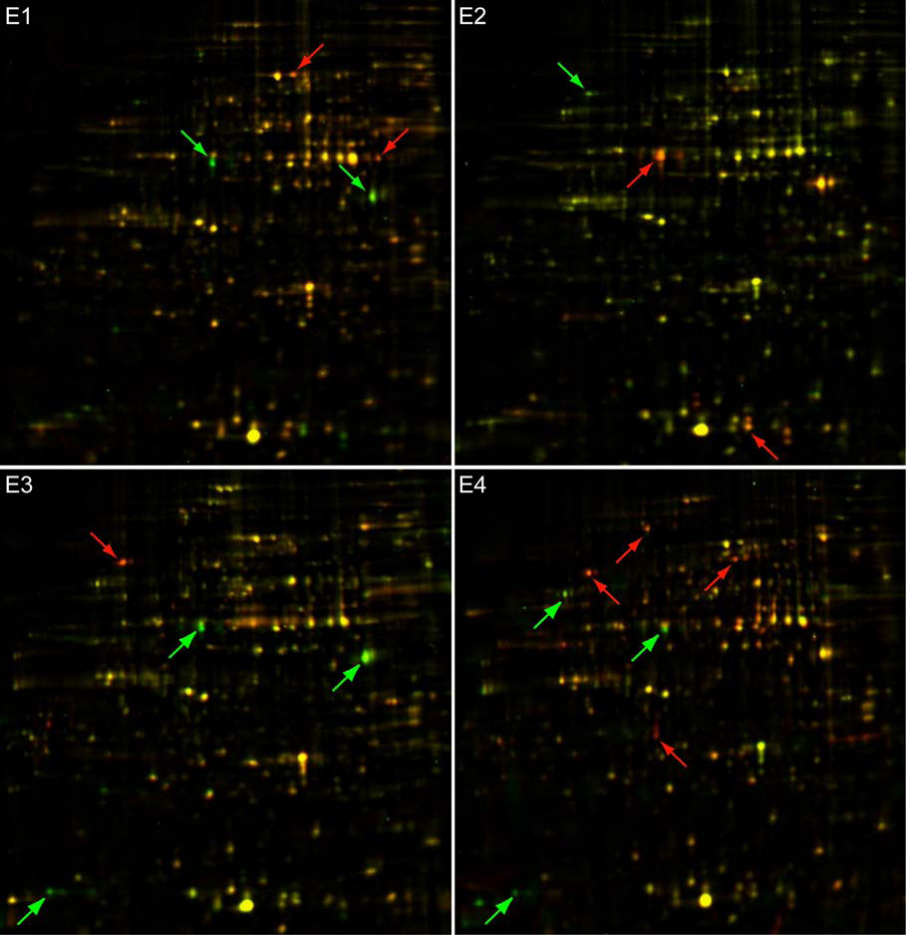
**2D gel merged Cy3/Cy5 representative images from conditions E1 to E4**. Gel images from the Cy3 control and Cy5 sample gels from each condition where superimposed and shown in one image. A general spot pattern is reproducibly seen in all gels; however, some proteins presented clear differential accumulations when compared to the Cy3 control gel. These proteins are highlighted by green (more abundant in the Cy3 control gel) and red (more abundant in the Cy5 sample gel) arrows.

**Figure 3:**
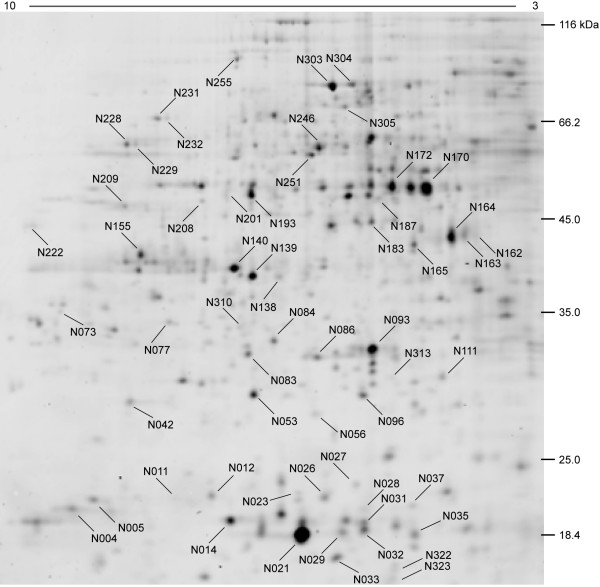
**Representative 2-D gel Cy5 stained proteins from the fruit mesocarp of E2 condition**. Spots that encompass a range between 15 and 116 kDa and had pI values spanning pH 3 to 10 are visible. Mass spectrometry identified spots are pointed out by their identifying tags.

2-D difference gel electrophoresis (DIGE) analysis was performed in order to identify changes in the amount of proteins present under different postharvest conditions. Figure 2 presents a representative image from Cy5/Cy3 overlapped gels of the four postharvest conditions under evaluation. A reproducible protein pattern is observed throughout the evaluated stages, and some changes in content can be visually detected. On average, the gels exhibited 560 spots, from which 242 well-focused spots were chosen for further analysis because they were present in all of the Cy3 reference gels. About 85% of these proteins presented a pI ranging from 5.5 to 8.0 and were between 18 to 65 kDa in size (Figure 3, Table 2). Some of the spots that were highly abundant or had a clear differential protein accumulation were identified by means of LC-MS/MS mass spectrometry (Table 2). The results of this analysis showed coverage of over 15% for most proteins. A complete list of the protein sequences with peptides delivered by mass spectra is presented in the additional file 2.

**Table 2:** Identified protein spots and their accumulation pattern

Spot No.^a^	Assignment^b^	Species	Accesion No.^c^	pI (Th/Exp)	MW (Th/Exp)	Mowse score^d^	coverage %	E1 vs E2	E1 vs E3	E1 vs E4	E2 vs E3	E2 vs E4	E3 vs E4
N004	Low molecular weight heat shock protein	[Malus × domestica]	AAF34133	5.4/8.0	18.2/20.0	149	14.4	**-1**	**1**	**0**	**1**	**1**	**-1**
N005	Glyceraldehyde-3-phosphate dehydrogenase	[Nicotiana tabacum]	CAB39974	7.7/7.9	36.7/21.1	408	27.3	**-1**	**-1**	**-1**	**1**	**1**	**0**
N011	Low molecular weight heat shock protein	[Malus × domestica]	AAF34133	5.4/7.3	18.2/21.2	155	25.0	**-1**	**0**	**-1**	**1**	**1**	**-1**
N012	ADP-ribosylation factor B1C	[Arabidopsis thaliana]	NP_186962	7.7/7.2	21.7/21.2	50	13.0	**1**	**-1**	**-1**	**-1**	**-1**	**0**
N014	ADP-ribosylation factor 1	[Solanum tuberosum]	CAA52468	6.8/7.1	22.6/19.6	316	26.9	0	0	0	0	0	0
N021	PRU1_PRUAV Major allergen Pru av 1	[Prunus avium]	O24248	5.9/6.6	16.7/18.4	189	50.0	0	0	0	0	0	0
N023	Small heat shock protein	[Retama raetam]	AAL32036	5.8/6.7	17.9/20.9	133	16.5	**-1**	**0**	**-1**	**1**	**1**	**-1**
N026	Peroxiredoxin	[Populus tremula × Populus tremuloides]	AAL90751	5.6/6.5	17.4/20.4	107	21.0	0	0	0	0	0	0
N027	Low molecular weight heat shock protein	[Malus × domestica]	AAF34133	5.4/6.3	18.2/22.0	258	25.0	**-1**	**-1**	**-1**	**1**	**1**	**0**
N028	Eukaryotic translation initiation factor 4D	[Medicago sativa]	CAA42065	5.5/6.3	17.7/19.7	119	13.7	0	0	0	0	0	0
N029	Major allergen Pru p 1	[Prunus persica]	ABB78006	5.8/6.4	17.6/19.0	530	45.5	**1**	**1**	**1**	**0**	**0**	**0**
N031	Small heat shock protein	[Prunus persica]	AAR99375	6.0/6.3	17.4/18.5	286	50.6	**-1**	**-1**	**-1**	**1**	**1**	**0**
N032	Small heat shock protein	[Prunus persica]	AAR99375	6.0/6.3	17.4/18.5	71	24.7	**-1**	**-1**	**-1**	**1**	**1**	**0**
N033	Superoxide dismutase	[Nicotiana plumbaginifolia]	CAA39444	5.6/6.4	15.4/17	134	17.1	0	0	0	0	0	0
N035	Thioredoxin H	[Prunus persica]	AAL26915	6.0/5.6	18.1/14.5	234	38.2	**-1**	**-1**	**-1**	**1**	**1**	**0**
N037	Low molecular weight heat shock protein	[Malus × domestica]	AAF34133	5.4/6.0	18.2/20.0	155	13.8	**-1**	**0**	**0**	**1**	**1**	**0**
N042	NADH dehydrogenase subunit F	[Graffenrieda latifolia]	CAJ84510	7.8/7.7	28.7/28.0	91	6.7	**-1**	**0**	**-1**	**1**	**1**	**-1**
N053	Manganese superoxide dismutase	[Camellia sinensis]	AAT68778	7.8/6.9	25.6/27.4	222	13.5	0	0	0	0	0	0
N056	Putative quinone reductase	[Vitis vinifera]	AAO12869	5.6/6.6	17.6/26.8	111	9.8	0	0	0	0	0	0
N073	Pectin methylesterase	[Nicotiana tabacum]	CAB57457	9.9/8.1	29.3/36.3	68	4.5	**-1**	**-1**	**0**	**-1**	**1**	**1**
N077	Porin	[Prunus armeniaca]	AAD38145	7.1/7.4	29.7/34.2	174	9.8	**1**	**0**	**1**	**-1**	**0**	**1**
N083	Triose phosphate isomerase cytosolic isoform	[Solanum chacoense]	AAR11379	5.7/6.9	27.0/30.6	111	15.4	0	0	0	0	0	0
N084	Proteasome subunit alpha type 7	[Cicer arietinum]	Q9SXU1	6.9/6.8	27.0/31.6	115	14.5	0	0	0	0	0	0
N086	Cytosolic ascorbate peroxidase	[Fragaria × ananassa]	AAB95222	5.7/6.5	27.2/30.2	231	16.0	0	0	0	0	0	0
N093	Abscisic stress ripening-like protein	[Prunus persica]	AAL26889	5.7/6.2	20.8/31.1	142	34.7	**0**	**0**	**1**	**-1**	**1**	**1**
N096	Quinone-oxidoreductase QR2	[Triphysaria versicolor]	AAG53945	6.4/6.3	22.1/27.4	102	37.6	0	0	0	0	0	0
N111	Iron-binding protein	[Pyrus pyrifolia]	ABD66595	5.4/5.8	19.5/29.3	116	11.3	**1**	**1**	**1**	**-1**	**-1**	**1**
N138	Annexin	[Medicago sativa]	CAA52903	5.4/6.7	35.0/38.7	88	13.0	**1**	**1**	**1**	**0**	**-1**	**0**
N139	Annexin	[Medicago sativa]	CAA52903	5.4/6.9	35.0/38.8	74	10.7	0	0	0	0	0	0
N140	NAD-dependent malate dehydrogenase	[Prunus persica]	AAL11502	6.6/7.0	35.5/39.5	390	21.4	0	0	0	0	0	0
N162	1-aminocyclopropane-1-carboxylate oxidase	[Prunus persica]	CAA54449	5.2/5.5	36.2/43.7	279	35.0	**0**	**-1**	**-1**	**1**	**0**	**-1**
N163	1-aminocyclopropane-1-carboxylate oxidase	[Prunus persica]	CAA54449	5.2/5.6	36.2/43.7	363	28.0	**-1**	**0**	**-1**	**1**	**1**	**-1**
N164	1-aminocyclopropane-1-carboxylate oxidase	[Prunus persica]	CAA54449	5.2/5.7	36.2/43.7	778	53.3	**-1**	**0**	**-1**	**1**	**1**	**-1**
N165	Oxidoreductase	[Arabidopsis thaliana]	NP_173786	8.5/5.9	41.0/42.6	224	10.4	**0**	**-1**	**-1**	**-1**	**-1**	**0**
N170	Actin	[Helianthus annuus]	AAF82805	5.6/5.8	41.7/49.2	979	45.4	0	0	0	0	0	0
N172	Actin	[Helianthus annuus]	AAF82805	5.6/6.0	41.7/48.7	358	17.0	0	0	0	0	0	0
N183	Alpha-1,4-glucan-protein synthase [UDP-forming]	[Pisum sativum]	O04300	5.7/6.2	41.6/45.4	2002	40.1	**0**	**0**	**-1**	**0**	**-1**	**-1**
N187	Anthocyanidin synthase	[Prunus persica]	BAC98347	5.4/6.1	31.1/47.2	287	24.5	**0**	**0**	**-1**	**0**	**-1**	**-1**
N193	Endopolygalacturonase	[Prunus persica]	AAC64184	6.2/6.9	41.3/48.9	389	46.1	**-1**	**0**	**-1**	**1**	**1**	**-1**
N201	NADP-dependent isocitrate dehydrogenase	[Prunus persica]	AAL11503	6.5/7.0	46.6/49.1	372	12.3	**-1**	**0**	**0**	**1**	**1**	**0**
N208	Glutamate Dehydrogenase 1	[Arabidopsis thaliana]	NP_197318	6.4/7.2	44.5/47.9	147	8.8	**0**	**-1**	**-1**	**-1**	**-1**	**0**
N209	Phosphoserine aminotransferase, chloroplast precursor	[Spinacia oleracea]	P52877	8.3/7.7	47.2/47.6	231	11.2	**0**	**0**	**-1**	**0**	**-1**	**-1**
N222	Quinone-oxidoreductase QR1	[Triphysaria versicolor]	AAG53944	9.4/8.2	34.9/46.1	158	9.4	**1**	**1**	**1**	**0**	**0**	**0**
N228	Catalase	[Prunus persica]	CAD42909	7.0/7.6	57.1/57.9	1356	51.8	**1**	**1**	**1**	**0**	**1**	**1**
N229	Catalase	[Prunus persica]	CAD42909	7.0/7.6	57.1/58.0	1151	40.4	**1**	**1**	**1**	**0**	**1**	**1**
N231	Dehydrin-like protein	[Prunus persica]	CAC00637	6.5/7.4	48.0/61.7	540	24.5	**-1**	**-1**	**-1**	**-1**	**-1**	**1**
N232	Dehydrin-like protein	[Prunus persica]	CAC00637	6.5/7.4	48.0/61.7	681	24.1	**0**	**-1**	**-1**	**-1**	**-1**	**0**
N246	UTP-glucose-1-phosphate uridylyltransferase	[Pyrus pyrifolia]	O64459	6.0/6.5	51.8/55.7	522	34.4	0	0	0	0	0	0
N251	ATPase subunit	[Beta vulgaris subsp. vulgaris]	CAA48650	5.7/6.5	55.0/54.8	645	30.6	0	0	0	0	0	0
N255	Pyruvate decarboxylase	[Fragaria × ananassa]	AAL37492	6.0/6.9	65.3/71.3	70	6.8	**-1**	**-1**	**-1**	**1**	**-1**	**-1**
N303	NADP-dependent malic enzyme	[Vitis vinifera]	P51615	6.1/6.4	65.2/65.4	600	16.4	0	0	0	0	0	0
N304	NADP-dependent malic enzyme	[Vitis vinifera]	P51615	6.1/6.3	65.2/65.6	291	6.3	**1**	**1**	**1**	**-1**	**0**	**1**
N305	Pyruvate decarboxylase	[Fragaria × ananassa]	AAL37492	6.0/6.3	65.3/61.8	473	18.5	**-1**	**0**	**-1**	**1**	**-1**	**-1**
N310	Thaumatin-like protein	[Prunus persica]	AAM00216	8.3/7.0	25.8/33.2	99	8.5	**0**	**0**	**-1**	**0**	**-1**	**-1**
N313	Glutathione S-transferase	[Cucurbita maxima]	BAC21261	7.7/6.1	25.0/28.3	102	6.0	**1**	**1**	**1**	**-1**	**-1**	**0**
N322	Putative glycine-rich RNA binding protein 1	[Catharanthus roseus]	AAF31402	8.7/6.1	14.2/16.4	40	11.0	**-1**	**-1**	**-1**	**-1**	**-1**	**0**
N323	Hypothetical protein	[Oryza sativa (japonica cultivar-group)]	BAD87001	11.6/6.1	17.4/15.7	46	4.5	**-1**	**-1**	**-1**	**-1**	**-1**	**0**

^a^: Spot numbers as assigned in Figure 3.

^b^: Protein assignment based on LC-MS/MS identification.

^c^: Gene bank accession number.

^d^: Probability that the match is random. Only proteins with scores greater than the minimum value for MS-based identification confidence are reported. Additional data about MS are reported in the additional file 2.

The values in the columns 9 to 14 indicate if the protein accumulation pattern changes between the conditions shown above them. Positive values indicate a higher abundance in the first condition whereas negative values indicate the opposite situation.

The theoretical isoelectric points (pI) and molecular weight (MW) of the proteins were calculated and compared to the experimental data. Most of the spots identified showed a good relationship to their theoretical MW (additional file 3). Only the dehydrin and the glyceraldehyde-3-phosphate dehydrogenase (GAPDH) showed some discrepancies; however, the experimental pI and molecular weight values for the dehydrin match quite well to those described by Wisniewski et al. [17]. Posttranslational modification, abnormal electrophoretic mobility or a smaller molecular weight for the *Prunus persica *GAPDH may explain the differences detected in that regard.

Some of the spots identified had matching protein IDs but different isoelectrical points and/or molecular weights (Table 2). In terms of their accumulation pattern, some displayed the same profile and others showed differences in their isoform. As Table 2 demonstrates, these spots include the following proteins: low molecular weight heat shock proteins, annexin, 1-aminocyclopropane-1-carboxylate oxidase, catalase, dehydrin, pyruvate dehydrogenase, actin and NADP-dependent malic enzyme. Overall, 58 distinct peach fruit mesocarp proteins were identified in this study.

### Multivariate analysis of the data through Hierarchical Clustering and PCA

In order to detect real changes in protein accumulation among the four postharvest conditions evaluated, the experimental data from 2-D gels were screened before being used for statistical analysis (see material and methods). A one-way analysis of variance (ANOVA) followed by a false discovery rate correction (FDR) allowed us to determinate that 43 -about 18% of the spots analyzed- showed statistically significant changes. Thirty-nine of these proteins could be detected and excised from the gels and then identified by mass spectrometry. Some of the identified proteins that changed during postharvest such as endopolygalacturonase (EndoPG) and the 1-aminocyclopropane-1-carboxylate (ACC oxidase) had been linked to the peach fruit ripening process [18,19]. Some, like dehydrin-like and thaumatin-like protein, had been detected in cold stressed plant tissues [20] (Table 2).

**Figure 4:**
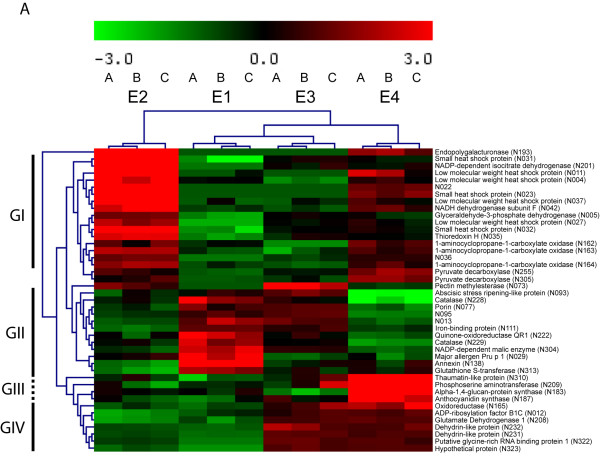
**Multivariate analysis of 2-D gel data**. Data from the forty three spots that showed a statistically significant change were hierarchically clustered using an average linkage algorithm and Euclidean distance as the distance metric. Four main groups were identified as indicated in the left side of the figure (GI-GIV). Proteins that have a similar accumulation pattern tend to cluster together.

A graphical display of the relationship between the differentially accumulated proteins was obtained using pairwise average-linkage cluster analysis, a technique that cluster together proteins with similar patterns of expression among different postharvest treatments [21]. Four general trends in the data could be visualized by this method, as displayed in Figure 4. The first group (GI) is represented by sixteen proteins that showed a pronounced increase during the normal softening process (E1-E2) compared to the absence of a noticeable increase in protein accumulation during the softening of cold stored fruit (E3-E4). This behaviour is exemplified by a group of proteins that cluster with a thioredoxin H, a protein that has an important role in the cellular redox regulation [22]. Group II showed twelve proteins that under the normal postharvest storage presented a decrease in their content between E1 and E2. An example of this is observed in the case of a catalase (N228), which showed its higher levels during E1 compared to E2 and E3, to decline even further in E4. Group III is represented by four proteins that have a pronounced accumulation only in the abnormal ripening that follows the low temperature storage (E4), like an anthocyanidin synthase and other proteins that cluster along with it. Group IV is composed by seven proteins that did not present any change between E1 and E2 fruit, whereas their level increased in E3, remaining constant in E4. One of those proteins is a dehydrin, which exhibit a clear high level in chilling stressed fruit (E3-E4) compared to healthy fruit (E1-E2). Some proteins did not fit in these four general clusters. For instance, two pyruvate decarboxylases (spots N255 and N305) were up-regulated in the softening process that leads to the development of chill injured fruit (transition from E3 to E4) more than in the normal ripening process (transition from E1 to E2). The protein pectin metilesterase (N073) also had a particular accumulation pattern, since it was up-regulated in the normal ripening process, whereas in the transition from E1 to E3 this increment was even higher. No other protein showed a similar trend. The EndoPG protein (spot N193) was also located in a different group, due to the degree of differences among its accumulation levels in the four postharvest conditions.

**Figure 5:**
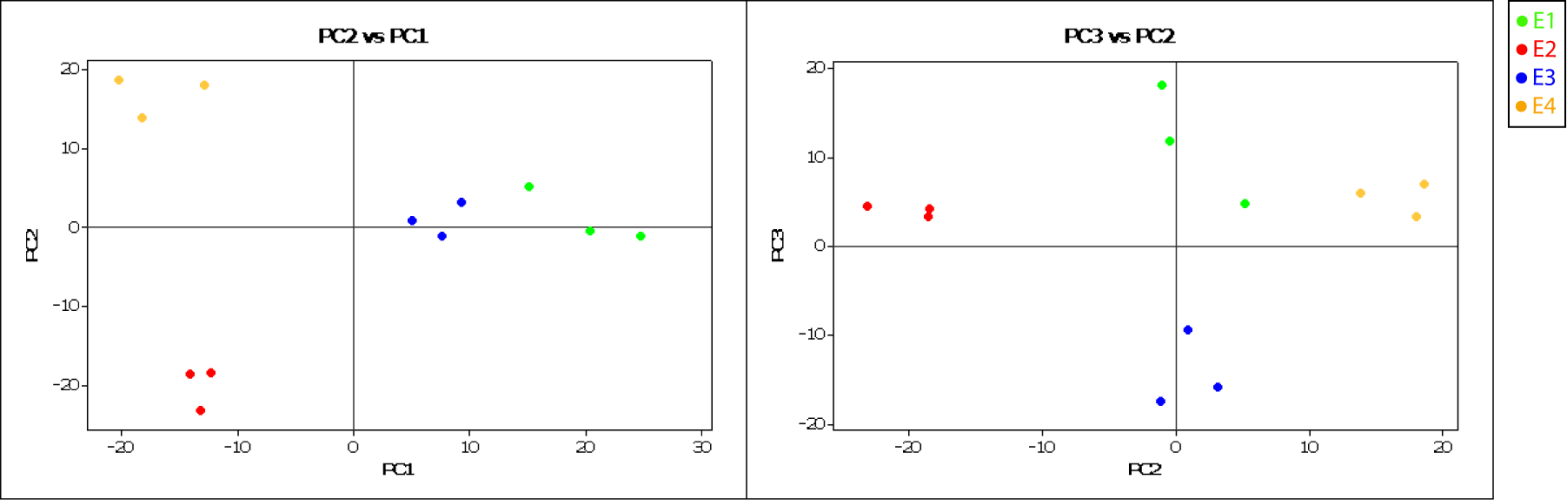
**Principal Component Analysis of the samples analyzed**. Principal component analysis score plots of first, second and third components are shown. It is possible to visualize how the samples can be separated by its ripeness condition (PC1), by their juice content (PC2) as well as by its exposure to cold temperatures (PC3). PCA has been performed on a covariance matrix. Each dot of the same colour represents a gel replicate.

A Principal Component Analysis (PCA) evaluation was performed in order to identify the most relevant features of the data set retrieved from the 2D-DIGE gels [23]. The PCA identifies new variables, called principal components, which are linear combinations of the original variables. These new features can describe a large amount of data in a lower dimensional space, which makes the PCA a useful tool for data categorization. The analysis of the weight loadings associated with each spot indicates which proteins have the greatest impact on the trends observed in the score plots (Figure 5). The first component was able to separate mature samples (E1 and E3) from ripe ones (E2 and E4). Endopolygalacturonase (N193), a thaumatin-like protein (N310) and a small heat shock protein (sHSP, N031) were among the proteins that had the higher impact in the ripe fruit classification. A catalase (N228) and an annexin (N138) were important for the trend observed for the mature fruit (additional file 3). The second component was able to differentiate chilling injured fruit samples (E4) from the ripe healthy fruit samples (E2). Again, the EndoPG (N193) and the sHSP (N031) had a high impact on the ripe healthy fruit trend, along with a NADP-dependent isocitrate dehydrogenase (N201). The proteins anthocyanidin synthase (N187), thaumatin-like protein (N310), alpha-1,4-glucan-protein synthase (N183) and phosphoserine aminotransferase (N209) were very relevant in differentiating chilling injured fruit from healthy fruit, which supports the results obtained by the hierarchical clustering. The third component differentiated between the fruit sample that was stored at low temperature (E3) and the mature fruit sample (E1) (Figure 5). A pectin methylesterase (N073) and a dehydrin-like protein (N232) played an important role in differentiating between the chilling injured fruit, while a Major allergen protein Pru p I (N029) displayed the opposite behavior. Interestingly, some proteins that were classified as not statistically differentially accumulated had a high impact on the PCA trends (additional file 3). This difference may be due to a false negative type II error. Finally, the three principal components represent nearly 56% of the original variance, with values of 25.9%, 19.3% and 11.3%, respectively, for the PC1, PC2 and PC3 components.

### Characterization of the differentially accumulated proteins

**Figure 6:**
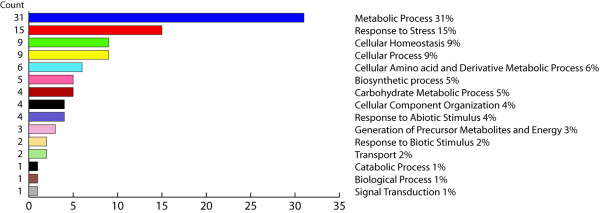
**Categories distribution of the identified differentially accumulated proteins**. The proteins that showed a statistically significant change were functionally sorted into 15 functional categories according to the Gene Ontology annotation. Counts indicate the number of hits that match each functional category.

In order to generate an overview of the most relevant biological processes involved in fruit softening and its response to the low temperature storage, a categorization of differentially accumulated proteins was performed based on the Gene Ontology (GO) annotation. This standardized categorization system provides a less subjective and more reproducible assignment of the biological processes underlying the chilling injury development [24]. Figure 6 displays the results obtained from the QuickGO tool. As expected, almost 25% of the proteins differentially expressed were associated with the biological processes 'response to stress' and 'cellular homeostasis'. The relevant impact detected in processes related to the metabolism of carbohydrates and amino acid metabolism (11% in total) is also noteworthy.

**Figure 7:**
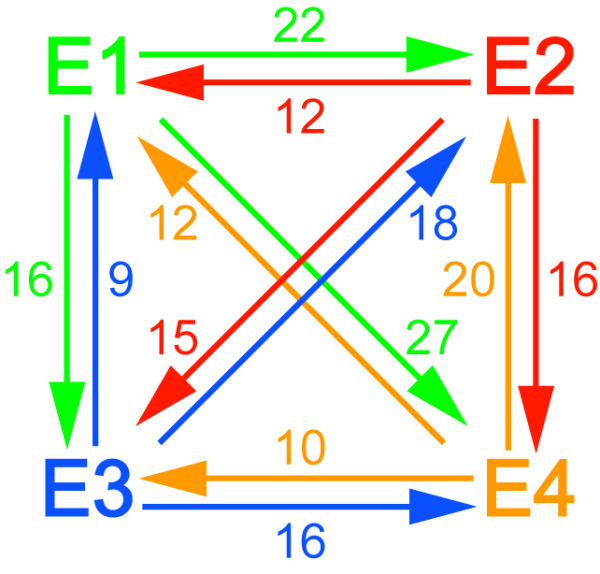
**Summary of the protein changes detected among the postharvest conditions evaluated**. The number of spots whose accumulation profile is altered is shown. As an example, 22 proteins are up-accumulated during the transition from stages E1 to E2 whereas 12 are down-regulated between these conditions.

**Figure 8:**
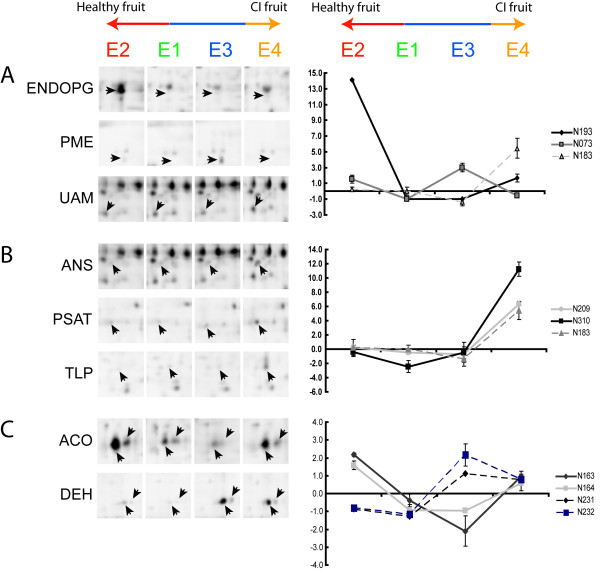
**Accumulation pattern of proteins differentially expressed in mature (E1), ripe (E2), cold stored (E3) and chilling injured (E4) post-harvest conditions**. Images from sections of two-dimensional gels are shown. Spots that have a clear differential accumulation pattern in some of the conditions, E1 to E4, are pointed by arrow heads. Their names and spot numbers follow the manuscript annotation. CI fruit - chilling injured fruit.

Figure 7 presents a summary of all the protein changes. The highest number of changes was detected between the E1 and E4 (39), and the smallest number was presented between E1 and E3 (25). These patterns correlate with those observed in the PCA score plots (Figure 5). The individual profiles of each protein are depicted in the additional file 3 and some of the most relevant changes detected are shown in Figure 8.

## Discussion

Peaches are climacteric fruit that display a decrease in mesocarp texture firmness. Melting varieties present a marked softening of this region. This process is less pronounced in non-melting varieties [25]. In *Prunus persica*, it forms part of the final stages of a broader phenomenon, the fruit ripening [26,27]. Of the physiological changes that the fruit displays during its ripening, the accumulation of soluble solids and changes in colour and aroma are very relevant for consumer acceptability. These attributes are severely affected in fruit that has been exposed to low temperatures for a prolonged amount of time [6]. This study identified several proteins that may be related to changes in fruit metabolism during cold storage and the subsequent ripening that leads to the development of chilling injury. These proteins are mainly linked to processes such as response to stress, cellular homeostasis, cellular component organization and carbohydrate metabolism (Figure 6). Our discussion will be guided by those general categories.

### Cellular component organization - Cell Wall Metabolism

The drastic changes in the texture of the fruit mesocarp in the melting varieties have been associated with a massive solubilization and depolymerization of pectin-derived polyuronides. These changes correlated with the concerted action of two proteins, endopolygalacturonases (EndoPG) and pectin methylesterases (PME) [28]. The main PME function is demethylation of the polyuronides so that they can be degraded by the EndoPG. Therefore, these proteins play an important role in dismantling the cell wall. Both the EndoPG and the PME are encoded by multi-gene families. Recently, Murayama et al. (2009) identified one member of each that is up-regulated above the other members during peach fruit softening, namely PpPG2 and PpPME2. PpPG2 is the EndoPG detected in our 2-D peach fruit gels and PpPME2 resembles the one we identified (N193 and N073, respectively; additional file 2). Additionally, they increase as we go from mature (E1) to ripe fruit (E2) (Table 2, Figure 8A). Several groups have reported changes in the degree of methylesterification and size of the homogalacturonans, which are localized to the middle lamella of the wall (Brummell et al. [18] and references therein). The PME levels that we detected were consistently remarkably higher at the end of cold storage (E3) than they were during normal fruit softening, whereas the levels of PpPG2 decreased to a minimum [18]. High PME activity during the cold storage could partially de-methylesterify homogalacturonans, which would maintain a high molecular weight due to the lack of EndoPG, protein that remains low in ripe chilling injured fruit (Figure 8A). It has been suggested that these large polymers are involved in the lack of juiciness characteristic of the mealy fruit [6].

It also has been reported that the arabinose (Ara) content of cell wall polysaccharides is different in chilling injured peaches [18]. Arabinose is mainly found in the cell wall Ara-containing polymers in the furanose ring configuration, though the substrate for this transfer (UDP-L-arabinofuranose) has not been found in plants [29]. Our results showed that accumulation of an α-1,4-glucan-protein synthase (N183), increased exclusively in chilling injured fruit (Figure 4 and Figure 8A). This protein is highly homologous to a rice UDP-arabinopyranose mutase (UAM), an enzyme that catalyzes the formation of UDP-L-arabinofuranose from UDP-L-arabinopyranose [30]. This increase suggests that this protein and the arabinans are linked to the development of chilling injury in *Prunus persica*. The results from the PCA (Figure 5, additional file 3) support the relevance of PpPG2, PME and UAM in the development of the chilling injury.

### Response to stress and cellular homeostasis

Fruit senescence can be considered as an oxidative phenomenon [31] that requires the presence of ROS (reactive oxygen species) in order to achieve a degradation state that will allow the seed release to occur. Several enzymes involved in ROS metabolism are expressed in early stages of fruit development and ripening and gradually are turned off as the fruit approaches senescence, making it more susceptible to be damaged by these molecules [31-34]. As shown in Figure 4, most of the proteins that are up-regulated in the mature fruit are related to ROS metabolism and show a decrease in their accumulation from mature to ripe fruit. After low temperature storage, their levels decrease in cold stored fruit (E3). As a result, the fruits' capacity to withstand cold induced stress is perturbed.

One of the most important enzymes involved in the control of ROS generation, particularly hydrogen peroxide, is catalase (CAT). At least two catalases have been identified in *P. persica *[35]. Until now, the only isoform detected in the 2-D gels is catalase 2 (spots N228 and N229, Table 2) [36], which appears in two spots with the same pI an different MW. Our results show that both spots are more abundant in firm fruit and decreased significantly in healthy soft fruit. A similar expression trend was found in apricot [37] and in tomato and saskatoon (*Amelanchier alnifolia *Nutt.) [38,39]. Peach fruit stored at 4°C for three weeks showed reduced catalase accumulation (Table 2). This result is supported by Wang et al. [40], who measured significantly lower CAT activity in peach fruit (*P. persica *cv. Okubao) stored at 0°C for 30 days as compared to controls. This negative effect on CAT accumulation could be harmful to the cold stored fruit due to the higher levels of H_2_O_2 _that could be present in their cells. The hydrogen peroxide would not only affect the redox status of the cell, but could influence signal transduction cascades, since this molecule has been postulated as a second messenger for signals generated by means of ROS [41-43].

The content of the iron binding protein ferritin (N111) changed in a manner that is similar to the catalase accumulation pattern. Interestingly, its biological function is also linked to the ROS metabolism, though its mechanism of action is somehow different. Ferritin can control the free iron concentration inside the cell and thus reduce its availability for the production of hydrogen peroxide derived hydroxyl radical [44]. Moreover, this protein has been shown to play a crucial role in the interaction between the iron homeostasis and oxidative stress in *Arabidopsis*, where its absence increased ROS levels as well as the activity of enzymes involved in their detoxification [45].

On the other hand, peach fruit that went soft without being exposed to cold storage accumulated small heat shock proteins. This seems to be a common ripening regulatory mechanism in fruit [46-48] as these proteins serve as protein chaperones and membrane stabilizers [48]. A low molecular weight heat shock protein (PpsHSP1) was identified in four spots with different molecular weight and pI. This protein shows an identity of 76% with carrot (*Daucus carota*) HSP, whereas a small heat shock protein (PpsHSP2) was found in two spots and shows an identity of 82% with a 17.4 kDa HSP protein from *Quercus suber*. Both proteins have been linked to the stabilization of cell components when facing stress conditions such as heat and oxidative stress [38,49,50]. The lower level of these proteins in the chill injured fruit would reduce their capacity to cope with oxidative stress.

The protein thioredoxin (TRX), which was found to increase during softening, plays an important role in cellular redox regulation [22]. TRXs are small, widely distributed proteins. Thioredoxin H (spot N035) has been associated with fruit ripening in several species, including peaches [51-53]. It has also been found to be responsive to abiotic stress stimuli such as chilling injury and drought [54,55]. Interestingly, both sHSPs [56] and the cGAPDH [57], which cluster together with the TRX (Figure 4), have been identified as established or potential targets of this protein.

One of the most abundant proteins in our gels was identified as an abscisic stress ripening-like protein (N093). It showed a slight, non-statistically significant increase between mature and ripe fruits and a clear down-regulation in mealy fruits (Table 2). This protein belongs to the ABA/WDS induced protein family (PFAM family ID PF02496) which is composed of plant proteins induced by water deficit stress or abscisic acid (ABA) stress and ripening. Frankel et al. [58] found that the banana ortholog presented a differential accumulation between two varieties with contrasting susceptibility to dehydration. It was highly accumulated in the tolerant one and virtually absent in the sensitive variety under normal conditions [59]. A similar situation was reported by Riccardi et al. in maize [60]. This protein may have protective function against dehydration in mature, ripe and cold stored fruit, though this protection is somehow diminished in mealy fruit. Further work must be done in order to determinate the function of this abundant protein in peach fruit and explain its decrease in chilling injured fruit.

The thaumatin-like protein (TLP) is an ethylene inducible protein. Its transcript has been shown to be highly expressed in overripe fruit and senescent flowers but not in senescent leaves [61]. This differential expression also has been reported in susceptible and chilled-injury resistant peach fruit varieties, although its relationship to this phenomenon was not established (Figure 8B, Table 2) [62].

### Response to abiotic stimulus

The 1-aminocyclopropane-1-carboxylate oxidase 1 (PpACO1; spots N162, N163 and N164; Table 2) is one of the main enzymes that control ethylene biosynthesis because it is responsible for converting 1-aminocyclopropane-1-carboxylate (ACC) into ethylene. Peach physiological measurements indicated that there were a good relationship among the ethylene levels and the ACO accumulation pattern at E1, E2 and E3, i.e., higher levels of ACO correlated with higher levels of ethylene. However, when peaches stored at low temperatures were allowed to ripen and became chill injured, the ethylene levels where significantly higher than they had been in the healthy fruit. This behaviour is similar to that which is observed in nectarine fruit ripened upon cold storage [63]. This ethylene increase did not correlate with PpACO1 content (Figure 8C). It is known that the main regulatory step in the ethylene synthesis in peaches is related to the *Prunus persica *1-aminocyclopropane-1-carboxylate synthase 1 (PpACS1) transcriptional and/or translational control [64]. However, we did not identify PpACS1 in this study and therefore cannot rule out the possibility that the ethylene increase observed in the E4 stage is associated to this protein. The posttranslational regulatory mechanisms that determine its presence in three different spots may help to explain this situation.

It is well known that dehydrins are induced by environmental stress factors like cold stress that result in cellular dehydration [65,66]. We found that a peach dehydrin-like protein (N228 and N229) was accumulated during the cold storage. This protein was also accumulated in the fruit that softened at room temperature after the exposure to cold. It is known that ethylene can induce the accumulation of dehydrins [67,68]. Given that ethylene is elevated in chilling injured fruit, it is likely that this could induce the increase of dehydrin (Figure 8C). The presence of another dehydrin (dehydrin2) in cold stored fruits also was reported [62]. Wisniewski et al. (2006) [66] demonstrated that both dehydrins had a different expression pattern in peach bark, leaf and embryo. However, no one has evaluated their behaviour in fruit mesocarp or the possibility that enhancing their presence in the fruit could improve or protect enzyme activities.

Another protein with increased accumulation during cold storage was a glutamate dehydrogenase (GDH). In their study of tobacco and grapevine, Skopelitis et al. [69] demonstrated that GDH accumulation was induced under ROS generating conditions such as high salt concentration and involved in the formation of glutamate, which in turn was used for proline biosynthesis, a molecule that could have a protective effect on cellular membranes [70]. GDH has also been implicated in cold tolerance [71] and associated with senescing organs. Ogundiwin et al. (2008) [62] using a transcriptomic approach detected an increase in the expression of a glutamate dehydrogenase in cold treated peach mesocarp tissue samples as compared to the control material, which is similar to the protein accumulation pattern detected in this publication. This pattern fits well with its stress responsive regulation (Table 2).

### Carbohydrate and amino acid metabolism

Cytosolic GAPDH (cGAPDH, N005) is a protein implicated in the conversion of glyceraldehyde 3-phosphate to 1,3-bisphosphoglycerate in the glycolytic pathway. An increase in this protein, which is affected in fruit stored at low temperatures, was detected during the transition from mature to ripe fruit. Experiments in cell suspension cultures of *A. thaliana *determined that these proteins may be an important target for hydrogen peroxide [72], being inactivated *in vitro *by H_2_O_2_. Thus, the authors suggested that cGAPDH also may play a role in mediating ROS signalling in plants.

NADP-isocitrate dehydrogenase (NADP-ICDH; N201) is another protein that presented a clear increment in its levels during fruit softening (transition from E1 to E2, Table 2). This protein catalyzes the conversion of isocitrate to 2-oxoglutarate, generating NADPH. It can be implicated in several processes such as the supply of NADPH, an important source of reducing power for the antioxidant fruit system [73]; the catabolism of organic acids in peach, with the concomitant decrease in the levels of organic acid content [74] and/or the degradation of stored carbon fuels in ripe fruit, such as sugars [27]; and the synthesis of 2-oxoglutarate, an organic acid involved in the coordination of the carbon-nitrogen metabolism [47]. Consequently, a decrease in this protein during the abnormal ripening that follows cold storage could have an important impact on the fruit's ability to withstand its metabolism as well as in its organoleptical characteristics. This statement is supported by the key role that it plays in separating healthy fruit from chilling injured ripe fruit in the PCA analysis (Figure 5, additional file 3).

The accumulation profile of NADP-dependent malic enzyme (NADP-ME, N304) is quite different from that of its counterpart NADP-isocitrate dehydrogenase. The former is preferentially expressed in mature fruit and down-regulated in ripe fruit. This profile fits with the one detected by Western Blot in peach fruit cv. Dixiland [27]. This protein, which uses malate to generate pyruvate and NADPH, may be relevant for the energy balance in the mature fruit [74]. This function would be affected in cold stored fruit (Table 2) and would constitute a source of stress.

The protein phosphoserine aminotransferase (PSAT; N209; Figure 8B) is preferentially accumulated in chilling injured fruit. It catalyzes the reversible formation of 2-oxoglutarate and phosphohydroxypyruvate to phosphoserine in the phosphorylated pathway of serine biosynthesis [75,76]. Therefore, it could be an important source of 2-oxoglutarate for a chilling injured fruit facing a down regulation of the NADP-ICDH accumulation. Nevertheless, instead of using the 2-oxoglutarate as a product, the enzyme could be helping the biosynthesis of serine. This amino acid is involved in the formation of compounds such as glycine betaine, which could have a beneficial effect on chilling stressed plants [76]. There is a need for more exhaustive studies of the metabolic effects of this enzyme on cold stressed fruit.

The accumulation pattern for the pyruvate decarboxylase (PDC) proteins (N255 and N305) also was detected by a microarray approach and activity assays in peach fruits [27,77]. During a process known as ethanolic fermentation, pyruvate is decarboxylated to acetaldehyde through the action of PDC. Thereafter, acetaldehyde is converted to ethanol by alcohol dehydrogenase (ADH). The ethanol and acetaldehyde generated could be used for the synthesis of ethyl esters, which are aroma constituents [77]. However, it has been reported that this fermentative pathway could have many others functions and be triggered by environmental stress like chilling [78]. Moyano et al. [79] showed that the strawberry fruit PDC1 responds to oxidative stress. Something similar could be present in chilling injured peach fruits [8], inducing a higher accumulation of the peach PDCs under this condition as compared to normal fruit. These higher levels of acetaldehyde could be beneficial for the cold stored fruit due to the inhibition of ethylene production [27]. The possibility of generating energy for the fruit through this pathway when mitochondrial respiration has been hampered may be crucial to the survival of chilling injured fruit [48].

### Generation of precursor metabolites and energy

Chloroplasts of higher plants contain the Ndh complex, which catalyzes the transfer of electrons from NADH to plastoquinone. This complex, together with electron draining reactions, protects plants against photo-oxidative-related stresses through the poise of the redox levels of the cyclic photosynthetic electron transporters [80]. During senescence, the expression of Ndh genes and the activity of the Ndh complex increase [81]. The higher levels of the subunit f (Ndhf, N042) of this complex in the ripe fruit are consistent with the developmental accumulation pattern. However, its deregulation, for example, by its over-expression, triggers the production of ROS, inducing programmed cell death in leaves [80]. Chloroplasts are present in green plant tissues and converted into chromoplasts during fruit ripening. In tomato (*Solanum lycopersicum*) and bell pepper (*Capsicum annuum*) fruit, chromoplasts accumulate both plastid encoded mRNAs and determined proteins at low levels, being translationally active [82]. The same situation seems to be present in peach fruit, which presents an active accumulation of this protein during ripening. It is plausible that levels of this protein, which is inserted in the thylakoid membrane, could have been affected by exposure to low temperatures, which are known to alter the cell membranes' conformation and structure [48]. This situation would lead to an imbalance in the natural senescence process in cold stored peach fruit and ripe cold injured fruit.

### Signal Transduction and Transport

An ADP-ribosylation factor (ARF; N012) also was accumulated during cold storage and ripening, suggesting that membrane trafficking may be altered during cold storage. ARFs work like GTP-dependent switches for the assembly/disassembly of the coat proteins driving vesicle budding/fusion [83]; however, they are linked to other processes such as alteration of membrane lipid compositions and act in remodelling various cell membrane compartments [84,85]. Changes in membrane composition and structure, with a concomitant increase in permeability, are among the first events triggered by chilling stress [48]; Efficient membrane trafficking therefore would be required in fruits under this condition [86]. During ripening, high levels of ROS also would damage the membrane. ARFs could be over-expressed under stress to counteract the continuous membrane fruit tissue harm.

Annexins are a diverse, multigene family of calcium-dependent, membrane-binding proteins that serve as targets for Ca^+2 ^in most eukaryotic cells. The main property of nearly all annexins is the binding to and possibly holding together of certain biological structures, especially membranes [87]. These proteins thus have the capacity to interact with lipid and protein moieties at the membranes, contributing to stress adaptation via regulation of various signalling pathways [88]. Due to its accumulation profile and the characteristics of these proteins, it may be involved in regulating stress-activated signalling pathways. This role is lost in the fruit which has been stored at low temperatures (Table 2).

### Other metabolic processes

The proteins that were up-regulated exclusively in fruit with chilling injury symptoms included an an anthocyanidin synthase (ANS; synonym LDOX - leucoanthocyanidin dioxygenase; N187; Figure 8B). This protein is one of the four dioxygenases (DOX) of the flavonoid biosynthetic pathway that has normally been associated with the conversion of leucoanthocyanidins to anthocyanidins [89], substrates for the synthesis of anthocyanin, a major pigment in fruit and flowers. Even though anthocyanidins are red in colour [90], under certain circumstances they can be enzymatically processed and derive into compounds that can be readily oxidized by polyphenol oxidases (PPO) and peroxidases (POD), yielding a brown colour [91,92]. Tissue browning is one of the symptoms associated to chilling injury and the 'O'Henry' variety is prone to it [18] (Table 1; additional file 1). However, the mechanism that causes this problem is still under investigation [6]. ANS accumulation in chilling injured fruit could enhance the synthesis of anthocyanidins in the fruit mesocarp and increase the substrates for POD and/or PPO. The recent identification of the ANS gene as the responsible for a QTL affecting browning in peaches, strongly supports our hypothesis [93]. It also strengthens the use of proteomics as a tool for the identification of markers that could potentially be useful in assisted breeding [55].

A glutathione S-transferase (GST; N313) was detected in the peach fruit 2-D gels and was preferentially expressed in mature (E1) fruit. This protein belongs to the tau subfamily, which is one of the four subfamilies that have been described. It is involved in cellular detoxification due to its ability to conjugate endobiotic and xenobiotic compounds to glutathione [94]. Its accumulation profile is quite similar to that of the annexin, which suggests fruit has active systems for coping with stress that run parallel to the action of CAT (N228 and N229). The down-regulation of these proteins and related metabolic mechanisms may be detrimental for fruit proper softening.

Changes in fruit ripening have been assessed mostly using transcriptomics [62,77,95]. However, some discrepancies have been found between transcript content analysis and proteins content analysis. A few proteomics analyses in peaches have been recently reported and their focus has been the analysis of fruit-pathogen interaction [27,36]. Obenland et al. [96] described the comparison between juicy and mealy fruit using 2-D analysis. They described five proteins as differentially accumulated between both stages. In addition, Borsani et al. [27] used 2-D DIGE to evaluate the protein changes during postharvest. They found 5 proteins that were differentially accumulated. Our study focused on the analysis of four postharvest stages including firm (E1), soft (E2), cold stored (E3) and chill-injured (E4) fruits. Therefore, we covered a broader set of postharvest conditions, enabling us to identify changes in a larger set of proteins. Thus, we got a deeper outlook of the molecular events that take place during softening and chilling injury.

## Conclusion

Using a comparative proteomic approach with 2-D DIGE allowed us to identify proteins that showed stage-specific changes in their accumulation pattern. Several proteins that are related to response to stress, cellular homeostasis, cellular component organization and carbohydrate metabolism were detected as being differentially accumulated. Finally, a significant proportion of the proteins identified had not been associated with softening, cold storage or chilling injury-altered fruit before; thus, comparative proteomics has proven to be a valuable tool for understanding fruit softening and postharvest.

## Methods

### Physiological parameters and experimental design

Large to medium size fruit from 'O'Henry' variety were harvest in the Aconcagua valley, Chile. Change in fruit ground color was considered to be a harvesting index. After selection, the peaches were transported to a packing facility for cooling. Fruit maturity and physiological parameters were measured at packing (E1), shelf-life at 20°C (E2), cold room storage (E3) and cold room storage plus shelf-life (E4). For further details please refer to Campos-Vargas et al [7].

### Protein extraction

Due to the significant biological variability expected from fruit harvested in the field, which is subjected to varying environmental conditions, four samples were pooled for each of the four conditions under evaluation. This approach should help reduce the inherent variation from the biological samples used in this study. About 0.5 g of mesocarp tissue from four fruits with similar physiological parameters were mixed and ground into a fine powder in liquid nitrogen using a mortar. The powder was mixed with 5 ml of protein extraction buffer containing sucrose 0.7 M, Tris base 0.5 M (pH 7.8), HCl 0.03 M, KCl 0.1 M, EDTA 0.005 M, DTT 0.002 M and PMSF 0.002 M, according to Abdi et al. [97]. A volume of 5.5 ml of Tris-saturated phenol pH 8.0 was added, and this phenol/aqueous solution was shaken vigorously for 5 min at room temperature. The phases were then separated by centrifugation at 8,500 × *g *for 14 min at 4°C. The phenol phase was recovered and re-extracted with an equal volume of protein extraction buffer and 1 ml of Tris-saturated phenol pH 8.0. The protein fraction was precipitated for two hours at -20°C by the addition of five volumes of 0.1 M ammonium acetate dissolved in methanol at -20°C. The precipitated material was collected by centrifugation at 8,500 × *g *for 12 min and the protein pellets were washed three times with cold ammonium acetate in methanol and once with 80% acetone at -20°C. The pellet was air dried at room temperature until the next day and solubilised in approximately 300 μl of buffer containing 7 M Urea, 2 M Thiourea, 0.4% ASB-14, 1.2% CHAPS, 10 mM Tris-HCl pH 8.5 (UTAC buffer). Samples were incubated at 25°C for 10 min and then pelleted by centrifugation at 12,000 × *g *for 15 min at room temperature in order to remove insoluble material. Protein concentration of these samples was determined by Bradford assay[98] using BSA as standard. Small amounts of sample were used for quantification to minimize interference of IEF compounds in the Bradford assay.

### 2-D DIGE experimental design

Equal amounts of protein extracted from the fruit pools from the four conditions evaluated were mixed and then covalently labelled with the Cy3 fluorophore. This sample was used as reference in all the gels run. Cy5 dye was used to label each of the four protein treatment pools separately. In order to ensure statistical significance for the data obtained, three gels were run for each treatment, yielding a total of 12 gels.

### Protein-cyanine dye labeling

Protein labeling was performed according to the manufacturer's recommended protocol (GE Healthcare/Amersham Biosciences). Cy3 and Cy5 N-hydroxysuccinamide (NHS) esters were freshly dissolved in anhydrous N,N-dimethylformamide (DMF, 99.8%, Sigma-Aldrich Chemie GmbH, Taufkirchen, Germany) and spun down for 30 seconds. The reference sample was covalently labelled with Cy3 fluorophore and samples from E1-E4 postharvest treatment were Cy5 labelled. In each case 50 μg of protein was labelled with 200 pmol of amine-reactive cyanine dyes. The labeling mixture was incubated on ice in the dark for 30 min and centrifuged at 12,000 × g for 5 min at room temperature. The reaction was terminated by adding 1 μl of 10 mM lysine (Sigma-Aldrich Chemie GmbH, Taufkirchen, Germany), and incubated on ice for 10 min. Cy3 labeled proteins were mixed with an equal amount of Cy5 labeled proteins and an equal volume of UTAC buffer plus 0.5% v/v ampholites 5-7 and 0.25% v/v ampholites 3-10. Finally, prior to IEF, samples were supplemented with 20 mM Dithiothreitol (DTT) and 2 mM Tris (2-Carboxyethyl) phosphine (TCEP) and buffer UTAC plus 0.25% v/v ampholites 5-7 and 0.12% v/v ampholites 3-10 to reach a final volume of 310 μl. All steps were performed at low light intensity.

### 2-D gel electrophoresis

Isoelectric focusing (IEF) was performed using a PROTEAN IEF Cell (Bio-Rad) and 17 cm length ReadyStrip IPG strips of pH range 3-10 NL (non-linear). A protein solution was loaded into IEF strip. After 20 minutes of incubation at room temperature, the strip was overlaid with mineral oil. Isoelectric focusing was undertaken using the following conditions: instrument temperature 20°C; maximum current limit 80 μA/strip; active rehydration at 50 V (volt) for 11 h; step 1, step-n-hold at 150 V for 25 minutes using rapid ramping; step 2, step-n-hold at 300 V for 60 minutes using rapid ramping; step 3, step-n-hold at 3,000 V for 3 h using normal ramping and step 4, step-n-hold at 8,000 V for 8 h using rapid ramping. On average, gels ran for 70,000 volt hours (Vh). After first dimension, proteins on the IPG strips were reduced and alkylated prior to electrophoresis by equilibrating the strip at room temperature for 10 min in Sodium Dodecyl Sulphate (SDS) equilibration buffer (375 mM Tris-HCl, pH 8.8, 6 M Urea, 20%(v/v) Glycerol and 2%(w/v) SDS) containing 2% (w/v) DTT followed by incubation for a further 10 min in SDS equilibration buffer containing 2.5% (w/v) Iodoacetamide instead of DTT. IPG strips were then sealed on top of SDS-PAGE 11-14.5% gradient self-cast gels (20 × 17 × 0.1 cm) with agarose plus bromophenol blue and run at 25 mA at 12°C using the Tris-Glycine-SDS buffer system until reach 3,000 Vh. Both IEF and SDS-PAGE gel were run in the dark.

### Gel imaging and spot detection

After SDS-PAGE, gels were screened using the Bio-Rad FX Phosphoimager. Cy3 images were scanned using a 532 nm internal laser and a 605DF50 emission filter and Cy5 images were scanned using a 635 nm laser and a 695DF55 emission filter. All gels were scanned at 100 μm (pixel size) resolution and images were stored at 150 dpi image size. Spot detection on 2D gel images was performed with version 3.3 of the Delta2D software package (Decodon GmbH, Greifswald, Germany). The settings used to detected spots were optimized using a representative Cy3 gel image and a number was assigned to each spot detected in the gel. The same parameters were used for the other gels and the spot identities were manually assigned to each spot in all the gels. Spot volumes were calculated with the Delta2D software and normalized to the corresponding image (relative expression of each spot compared to the total spot volume of the image) and against the pooled Cy3 standard of each individual spot (ratio of one particular sample spot volume to the corresponding Cy3 spot volume). The resulting values were exported to an excel spreadsheet.

### Statistical analysis

Only spots that were present in all of the Cy3 images were used for further statistical analysis. If a spot was recovered in at least two of three gel images for a given condition and was present in all replicates of the rest of the postharvest treatment, the missing value was replaced with the mean of the two existing values for that protein spot in that condition. In order to identify protein changes among the four different postharvest stages, data was first subjected to a transformation that stabilizes the variance across the full range of values [99,100], and then standardized using a robust Z score [101]. One-way analysis of variance (ANOVA) was then executed using MeV v4.0 software, which allows for simultaneous control for false discovery rate (FDR), which is desired when multiple testing procedures are performed [102]. Thus, ANOVA was performed with a false discovery procedure that minimized the number of false positives to less than one, followed by Tukey's test. A significance p-value cut-off of 0.05 was used to determinate differentially accumulated proteins. Standard hierarchical clustering with average linkage (Euclidean distance) was used to cluster these proteins. PCA analysis was carried out in parallel to this using Minitab software version 14 and was performed on a covariance matrix.

### Identification of proteins by mass spectrometry

Spots were excised from colloidal coomassie stained gels. Spots trypsinization and LC-MS/MS analyses were performed as follows. In gel digestion was performed using a MassPrep station (Perkin Elmer, USA). Proteins within gel slices were first reduced and alkylated using DTT and Iodoacetamide respectively and then digested to peptides by porcine trypsin (Promega, USA). Resultant peptides were eluted from the gel pieces in 15 μl of 0.1% formic acid. Five microliters of this solution were injected onto a reverse phase column (15 cm 75 μm internal diameter C18 PepMap column) coupled to an Eksigent nano LC system (Presearch, UK) at a flow rate of 300 nl/min attached to a LTQ Orbitrap (Thermo). The LTQ Orbitrap was operated in data dependant mode, MS followed by 2 × MS/MS with a dynamic exclusion of 1.5 Da (m/z values). Resulting data files were converted to .mgf file format using Bioworks version 3.2 (Thermo, USA) and searched against the IPI_mouse database (51,326 entries) using MASCOT version 2.2 (Matrix Science, London, UK) with the following parameter settings: 2 miscleavages, variable methioine oxidation, carboxymethyl cysteine fixed modification, fragment mass tolerance of 0.8 Da, peptide tolerance of 1.0 Da. Probability-based MASCOT scores were used to evaluate identifications. Only scores considered significant by MASCOT (confidence greater than 95%) were contemplated for further analysis. The peptides obtained from mass spectrometry were used to carry out TblastN of the *Prunus persica *contigs generated [103]. These sequences were further translated into aminoacidic sequences in order to be able to establish homology comparisons against proteins reported in the literature.

### Gene ontology annotation

Biological Processes integrated in the Plant GO slim (goslim_plant), a limited subset of high-level GO terms extracted from the whole Gene Ontology, were used for this analysis. The UniProtKB accession numbers of each differentially accumulated protein were retrieved from the UniProtKB database and loaded onto the QuickGO annotation page [24]. In total, 30 accession numbers were loaded, 25 of which had a GO Biological Process Annotation assigned (additional file 4). Spots N138, N255, N305, N313, N322 and N323 did not have an annotation. The results of this procedure were used to perform the functional analysis of the other proteins.

## Abbreviations

2-D DIGE: two-dimensional difference gel electrophoresis; ACC oxidase: 1-aminocyclopropane-1-carboxylate oxidase; ADH: alcohol dehydrogenase; ARF: ADP-ribosylation factor; ANOVA: one-way analysis of variance; ANS: anthocyanidin synthase; Ara: arabinose; CAT: catalase; cGAPDH: cytosolic glyceraldehyde-3-phosphate dehydrogenase; Da: Dalton; DMF: N,N-dimethylformamide; DTT: Dithiothreitol; EndoPG: endopolygalacturonase; FDR: false discovery rate correction; GAPDH: glyceraldehyde-3-phosphate dehydrogenase; GDH: glutamate dehydrogenase; IEF: Isoelectric focusing; kDa: kilodalton; LDOX: leucoanthocyanidin dioxygenase; Min: minute; mM: milimolar; MW: molecular weight; N: Newton; NADP-ICDH: NADP-isocitrate dehydrogenase; NADP-ME: NADP-dependend malic enzyme; Ndhf: NADH dehydrogenase subunit F; NL: non-linear; pI: isoelectrical point; PME: pectin methylesterase; pmol: picomole; POD: peroxidases; PpACO1: *Prunus persica *1-aminocyclopropane-1-carboxylate oxidase 1; PpACS1: *Prunus persica *1-aminocyclopropane-1-carboxylate synthase 1; PpPG2: *Prunus persica *polygalacturonase 2; PPO: polyphenol oxidases; PpsHSP1: *Prunus persica *small heat shock protein 1; PpsHSP2: *Prunus persica *small heat shock protein 2; PSAT: phosphoserine aminotransferase; ROS: reactive oxygen species; sHSP: small heat shock protein; SDS: Sodium Dodecyl Sulphate; TLP: thaumatin-like protein; TCEP: Tris(2-Carboxyethyl) phosphine; TRX: thioredoxin; UAM: UDP-arabinopyranose mutase; V: volt; Vh: volt hours; μA: microampere; μl: microliters; μm: micrometers.

## Authors' contributions

RN, performed the experiments and analysis of the data; CS, performed some of the experiments; KL, was involved in the protein sequencing and the set up of DIGE. RB-Y, RC-V, MG, LM, JR, HS and AO supervised the Chilean Functional Genomics Consortium in Nectarines which provided the platform and tools for this proteomic analysis. They were also involved in the discussion of the experimental model and data analysis. All authors read and approved the manuscript.

## Supplementary Material

Additional file 1 **Evaluation of the mesocarp browning degree among fruits from the four different postharvest conditions evaluated**. Fruit mesocarp images from three representative samples from the E1, E2, E3 and E4 conditions are shown. The degree of browning was assessed visually and values were assigned to each condition using E1 as standard. As can be seem, the fruits from the E4 condition have a darker brown mesocarp color when compared to the other four conditions. + no browning; ++ no browning degree with a color change of the mesocarp; +++ medium browning degree; ++++ high browning degree. Click here for file

Additional file 2 **Peptide sequences obtained by mass spectrometry and their localization within proteins**. A compendium of the protein sequences containing the peptide sequences (highlighted) delivered by the mass spectra is presented. Click here for file

Additional file 3 **Differential accumulation of each spot detected in the 2-D gels analysis from peach fruit mesocarp**. The experimental values of isolelectrical point (pI) and molecular weight (MW) of all the spots analyzed, as well as its accumulation pattern and the PCA weight loadings values are listed. Click here for file

Additional file 4 **Gene Ontology Annotation**. A detailed description of each biological process assigned to the differentially accumulated proteins is listed together with the evidence source used by the GO annotation system. Click here for file

## References

[B1] Giovannoni J Molecular Biology of Fruit Maturation and Ripening Annu Rev Plant Physiol Plant Mol Biol 2001 52 725 749 10.1146/annurev.arplant.52.1.725 11337414

[B2] Carrari F Baxter C Usadel B Urbanczyk-Wochniak E Zanor MI Nunes-Nesi A Nikiforova V Centero D Ratzka A Pauly M Integrated analysis of metabolite and transcript levels reveals the metabolic shifts that underlie tomato fruit development and highlight regulatory aspects of metabolic network behavior Plant Physiol 2006 142 4 1380 1396 10.1104/pp.106.088534 17071647 PMC1676044

[B3] White PJ Recent advances in fruit development and ripening: an overview J Exp Bot 2002 53 377 1995 2000 10.1093/jxb/erf105 12324524

[B4] Brummell DA Cell wall disassembly in ripening fruit Funct Plant Biol 2006 33 2 103 119 10.1071/FP05234 32689218

[B5] Lester DR Speirs J Orr G Brady CJ Peach (Prunus persica) endopolygalacturonase cDNA isolation and mRNA analysis in melting and nonmelting peach cultivars Plant Physiol 1994 105 1 225 231 10.1104/pp.105.1.225 8029352 PMC159349

[B6] Lurie S Crisosto CH Chilling injury in peach and nectarine Postharvest Biol Tec 2005 37 3 195 208 10.1016/j.postharvbio.2005.04.012

[B7] Campos-Vargas R Becerra O Baeza-Yates R Cambiazo V Gonzalez M Meisel L Orellana A Retamales J Silva H Defilippi BG Seasonal variation in the development of chilling injury in 'O'Henry' peaches Sci Hortic-Amsterdam 2006 110 1 79 83 10.1016/j.scienta.2006.06.019

[B8] Wang LJ Chen SJ Kong WF Li SH Archbold DD Salicylic acid pretreatment alleviates chilling injury and affects the antioxidant system and heat shock proteins of peaches during cold storage Postharvest Biol Tec 2006 41 3 244 251 10.1016/j.postharvbio.2006.04.010

[B9] Imahori Y Takemura M Bai J Chilling-induced oxidative stress and antioxidant responses in mume (Prunus mume) fruit during low temperature storage Postharvest Biol Tec 2008 49 1 54 60 10.1016/j.postharvbio.2007.10.017

[B10] Zhu SH Zhou J Effects of nitric oxide on fatty acid composition in peach fruits during storage J Agr Food Chem 2006 54 25 9447 9452 10.1021/jf062451u 17147431

[B11] Zhang C Tian S Crucial contribution of membrane lipids' unsaturation to acquisition of chilling-tolerance in peach fruit stored at 0°C Food Chemistry 2009 115 2 405 411 10.1016/j.foodchem.2008.12.021

[B12] Carpentier SC Coemans B Podevin N Laukens K Witters E Matsumura H Terauchi R Swennen R Panis B Functional genomics in a non-model crop: transcriptomics or proteomics? Physiol Plantarum 2008 133 2 117 130 10.1111/j.1399-3054.2008.01069.x 18312499

[B13] Lee DG Ahsan N Lee SH Lee JJ Bahk JD Kang KY Lee BH Chilling stress-induced proteomic changes in rice roots J Plant Physiol 2009 166 1 1 11 10.1016/j.jplph.2008.02.001 18433929

[B14] Rose JKC Bashir S Giovannoni JJ Jahn MM Saravanan RS Tackling the plant proteome: practical approaches, hurdles and experimental tools Plant J 2004 39 5 715 733 10.1111/j.1365-313X.2004.02182.x 15315634

[B15] Lilley KS Dupree P Methods of quantitative proteomics and their application to plant organelle characterization J Exp Bot 2006 57 7 1493 1499 10.1093/jxb/erj141 16617121

[B16] Unlu M Morgan ME Minden JS Difference gel electrophoresis: a single gel method for detecting changes in protein extracts Electrophoresis 1997 18 11 2071 2077 10.1002/elps.1150181133 9420172

[B17] Wisniewski M Webb R Balsamo R Close TJ Yu XM Griffith M Purification, immunolocalization, cryoprotective, and antifreeze activity of PCA60: A dehydrin from peach (Prunus persica) Physiol Plantarum 1999 105 4 600 608 10.1034/j.1399-3054.1999.105402.x

[B18] Brummell DA Dal Cin V Lurie S Crisosto CH Labavitch JM Cell wall metabolism during the development of chilling injury in cold-stored peach fruit: association of mealiness with arrested disassembly of cell wall pectins J Exp Bot 2004 55 405 2041 2052 10.1093/jxb/erh228 15310820

[B19] Ruperti B Bonghi C Rasori A Ramina A Tonutti P Characterization and expression of two members of the peach 1-aminocyclopropane-1-carboxylate oxidase gene family Physiol Plant 2001 111 3 336 344 10.1034/j.1399-3054.2001.1110311.x 11240918

[B20] Renaut J Hausman JF Wisniewski ME Proteomics and low-temperature studies: bridging the gap between gene expression and metabolism Physiol Plantarum 2006 126 1 97 109 10.1111/j.1399-3054.2006.00617.x

[B21] Sokal RR Michener CD A statistical method for evaluating systematic relationships Univ Kansas Sci Bull 1958 38 2 1409 1438

[B22] Meyer Y Reichheld JP Vignols F Thioredoxins in Arabidopsis and other plants Photosynth Res 2005 86 3 419 433 10.1007/s11120-005-5220-y 16307307

[B23] Ringner M What is principal component analysis? Nat Biotechnol 2008 26 3 303 304 10.1038/nbt0308-303 18327243

[B24] Barrell D Dimmer E Huntley RP Binns D O'Donovan C Apweiler R The GOA database in 2009--an integrated Gene Ontology Annotation resource Nucleic Acids Res 2009 37 Database D396 403 10.1093/nar/gkn803 18957448 PMC2686469

[B25] Morgutti S Negrini N Nocito FF Ghiani A Bassi D Cocucci M Changes in endopolygalacturonase levels and characterization of a putative endo-PG gene during fruit softening in peach genotypes with nonmelting and melting flesh fruit phenotypes New Phytol 2006 171 2 315 328 10.1111/j.1469-8137.2006.01763.x 16866939

[B26] Trainotti L Bonghi C Ziliotto F Zanin D Rasori A Casadoro G Ramina A Tonutti P The use of microarray mu PEACH1.0 to investigate transcriptome changes during transition from pre-climacteric to climacteric phase in peach fruit Plant Sci 2006 170 3 606 613 10.1016/j.plantsci.2005.10.015

[B27] Borsani J Budde CO Porrini L Lauxmann MA Lombardo VA Murray R Andreo CS Drincovich MF Lara MV Carbon metabolism of peach fruit after harvest: changes in enzymes involved in organic acid and sugar level modifications J Exp Bot 2009 60 6 1823 1837 10.1093/jxb/erp055 19264753

[B28] Murayama H Arikawa M Sasaki Y Dal Cin V Mitsuhashi W Toyomasu T Effect of ethylene treatment on expression of polyuronide-modifying genes and solubilization of polyuronides during ripening in two peach cultivars having different softening characteristics Postharvest Biol Tec 2009 52 2 196 201 10.1016/j.postharvbio.2008.11.003

[B29] Harholt J Jensen JK Sorensen SO Orfila C Pauly M Scheller HV ARABINAN DEFICIENT 1 is a putative arabinosyltransferase involved in biosynthesis of pectic arabinan in Arabidopsis Plant Physiol 2006 140 1 49 58 10.1104/pp.105.072744 16377743 PMC1326030

[B30] Konishi T Takeda T Miyazaki Y Ohnishi-Kameyama M Hayashi T O'Neill MA Ishii T A plant mutase that interconverts UDP-arabinofuranose and UDP-arabinopyranose Glycobiology 2007 17 3 345 354 10.1093/glycob/cwl081 17182701

[B31] Qin G Meng X Wang Q Tian S Oxidative damage of mitochondrial proteins contributes to fruit senescence: a redox proteomics analysis J Proteome Res 2009 8 5 2449 2462 10.1021/pr801046m 19239264

[B32] Brennan T Rychter A Frenkel C Activity of Enzymes Involved in the Turnover of Hydrogen-Peroxide During Fruit Senescence Botanical Gazette 1979 140 4 384 388 10.1086/337101

[B33] Wang SY Jiao H Changes in oxygen-scavenging systems and membrane lipid peroxidation during maturation and ripening in blackberry J Agric Food Chem 2001 49 3 1612 1619 10.1021/jf0013757 11312904

[B34] Mondal K Sharma NS Malhotra SP Dhawan K Singh R Antioxidant systems in ripening tomato fruits Biol Plant 2004 48 1 49 53 10.1023/B:BIOP.0000024274.43874.5b

[B35] Bagnoli F Danti S Magherini V Cozza R Innocenti AM Racchi ML Molecular cloning, characterisation and expression of two catalase genes from peach Funct Plant Biol 2004 31 4 349 357 10.1071/FP03203 32688905

[B36] Chan Z Qin G Xu X Li B Tian S Proteome approach to characterize proteins induced by antagonist yeast and salicylic acid in peach fruit J Proteome Res 2007 6 5 1677 1688 10.1021/pr060483r 17381148

[B37] Grimplet J Romieu C Audergon JM Marty I Albagnac G Lambert P Bouchet JP Terrier N Transcriptomic study of apricot fruit (Prunus armeniaca) ripening among 13 006 expressed sequence tags Physiol Plantarum 2005 125 3 281 292 10.1111/j.1399-3054.2005.00563.x

[B38] Rogiers SY Kumar GNM Knowles NR Maturation and ripening of fruit of Amelanchier alnifolia Nutt. are accompanied by increasing oxidative stress Ann Bot 1998 81 2 203 211 10.1006/anbo.1997.0543

[B39] Jimenez A Creissen G Kular B Firmin J Robinson S Verhoeyen M Mullineaux P Changes in oxidative processes and components of the antioxidant system during tomato fruit ripening Planta 2002 214 5 751 758 10.1007/s004250100667 11882944

[B40] Wang YS Tian SP Xu Y Effects of high oxygen concentration on pro- and anti-oxidant enzymes in peach fruits during postharvest periods Food Chemistry 2005 91 1 99 104 10.1016/j.foodchem.2004.05.053

[B41] Yang T Poovaiah BW Hydrogen peroxide homeostasis: activation of plant catalase by calcium/calmodulin Proc Natl Acad Sci USA 2002 99 6 4097 4102 10.1073/pnas.052564899 11891305 PMC122654

[B42] Desikan R S AH-M Hancock JT Neill SJ Regulation of the Arabidopsis transcriptome by oxidative stress Plant Physiol 2001 127 1 159 172 10.1104/pp.127.1.159 11553744 PMC117972

[B43] Apel K Hirt H Reactive oxygen species: metabolism, oxidative stress, and signal transduction Annu Rev Plant Biol 2004 55 373 399 10.1146/annurev.arplant.55.031903.141701 15377225

[B44] Deak M Horvath GV Davletova S Torok K Sass L Vass I Barna B Kiraly Z Dudits D Plants ectopically expressing the iron-binding protein, ferritin, are tolerant to oxidative damage and pathogens Nat Biotechnol 1999 17 2 192 196 10.1038/6198 10052358

[B45] Ravet K Touraine B Boucherez J Briat JF Gaymard F Cellier F Ferritins control interaction between iron homeostasis and oxidative stress in Arabidopsis Plant J 2008 18826427 10.1111/j.1365-313X.2008.03698.x

[B46] Bianco L Lopez L Scalone AG Di Carli M Desiderio A Benvenuto E Perrotta G Strawberry proteome characterization and its regulation during fruit ripening and in different genotypes J Proteomics 2009 72 4 586 607 10.1016/j.jprot.2008.11.019 19135558

[B47] Faurobert M Mihr C Bertin N Pawlowski T Negroni L Sommerer N Causse M Major proteome variations associated with cherry tomato pericarp development and ripening Plant Physiol 2007 143 3 1327 1346 10.1104/pp.106.092817 17208958 PMC1820912

[B48] Sevillano L Sanchez-Ballesta MT Romojaro F Flores FB Physiological, hormonal and molecular mechanisms regulating chilling injury in horticultural species. Postharvest technologies applied to reduce its impact J Sci Food Agric 2009 89 4 555 573 10.1002/jsfa.3468

[B49] Ahn YJ Zimmerman JL Introduction of the carrot HSP17.7 into potato (Solanum tuberosum L.) enhances cellular membrane stability and tuberization in vitro Plant Cell Environ 2006 29 1 95 104 10.1111/j.1365-3040.2005.01403.x 17086756

[B50] Pla M Huguet G Verdaguer D Puigderrajols P Llompart B Nadal A Molinas M Stress proteins co-expressed in suberized and lignified cells and in apical meristems Plant Sci 1998 139 1 49 57 10.1016/S0168-9452(98)00169-1

[B51] Callahan AM Morgens PH Cohen RA Isolation and Initial Characterization of Cdnas for Messenger-Rnas Regulated During Peach Fruit-Development J Am Soc Hortic Sci 1993 118 4 531 537

[B52] Manrique-Trujillo SM Ramirez-Lopez AC Ibarra-Laciette E Gomez-Lim MA Identification of genes differentially expressed during ripening of banana J Plant Physiol 2007 164 8 1037 1050 10.1016/j.jplph.2006.07.007 16934912

[B53] Levi A Davis A Hernandez A Wechter P Thimmapuram J Trebitsh T Tadmor Y Katzir N Portnoy V King S Genes expressed during the development and ripening of watermelon fruit Plant Cell Rep 2006 25 11 1233 1245 10.1007/s00299-006-0163-0 16802118

[B54] Yan SP Zhang QY Tang ZC Su WA Sun WN Comparative proteomic analysis provides new insights into chilling stress responses in rice Mol Cell Proteomics 2006 5 3 484 496 16316980 10.1074/mcp.M500251-MCP200

[B55] Hajheidari M Eivazi A Buchanan BB Wong JH Majidi I Salekdeh GH Proteomics uncovers a role for redox in drought tolerance in wheat J Proteome Res 2007 6 4 1451 1460 10.1021/pr060570j 17343403

[B56] Buchanan BB Balmer Y Redox regulation: A broadening horizon Annu Rev Plant Biol 2005 56 187 220 10.1146/annurev.arplant.56.032604.144246 15862094

[B57] Yamazaki D Motohashi K Kasama T Hara Y Hisabori T Target proteins of the cytosolic thioredoxins in Arabidopsis thaliana Plant Cell Physiol 2004 45 1 18 27 10.1093/pcp/pch019 14749482

[B58] Frankel N Carrari F Hasson E Iusem ND Evolutionary history of the Asr gene family Gene 2006 378 74 83 10.1016/j.gene.2006.05.010 16822623

[B59] Carpentier SC Witters E Laukens K Van Onckelen H Swennen R Panis B Banana (Musa spp.) as a model to study the meristem proteome: Acclimation to osmotic stress Proteomics 2007 7 1 92 105 10.1002/pmic.200600533 17149779

[B60] Riccardi F Gazeau P Jacquemot MP Vincent D Zivy M Deciphering genetic variations of proteome responses to water deficit in maize leaves Plant Physiol Biochem 2004 42 12 1003 1011 10.1016/j.plaphy.2004.09.009 15707837

[B61] Ruperti B Cattivelli L Pagni S Ramina A Ethylene-responsive genes are differentially regulated during abscission, organ senescence and wounding in peach (Prunus persica) J Exp Bot 2002 53 368 429 437 10.1093/jexbot/53.368.429 11847241

[B62] Ogundiwin EA Marti C Forment J Pons C Granell A Gradziel TM Peace CP Crisosto CH Development of ChillPeach genomic tools and identification of cold-responsive genes in peach fruit Plant Mol Biol 2008 68 4-5 379 397 10.1007/s11103-008-9378-5 18661259

[B63] Manganaris GA Vasilakakis M Diamantidis G Mignani I Changes in cell wall neutral sugar composition and ethylene evolution as potential indicators of woolliness in cold-stored nectarine fruit J Food Qual 2005 28 5-6 407 416 10.1111/j.1745-4557.2005.00044.x

[B64] Tatsuki M Haji T Yamaguchi M The involvement of 1-aminocyclopropane-1-carboxylic acid synthase isogene, Pp-ACS1, in peach fruit softening J Exp Bot 2006 57 6 1281 1289 10.1093/jxb/erj097 16531466

[B65] Close TJ Dehydrins: A commonality in the response of plants to dehydration and low temperature Physiol Plantarum 1997 100 2 291 296 10.1111/j.1399-3054.1997.tb04785.x

[B66] Wisniewski ME Bassett CL Renaut J Farrell R Jr Tworkoski T Artlip TS Differential regulation of two dehydrin genes from peach (Prunus persica) by photoperiod, low temperature and water deficit Tree Physiol 2006 26 5 575 584 16452071 10.1093/treephys/26.5.575

[B67] Richard S Morency MJ Drevet C Jouanin L Seguin A Isolation and characterization of a dehydrin gene from white spruce induced upon wounding, drought and cold stresses Plant Mol Biol 2000 43 1 1 10 10.1023/A:1006453811911 10949369

[B68] Yu XM Griffith M Wiseman SB Ethylene induces antifreeze activity in winter rye leaves Plant Physiol 2001 126 3 1232 1240 10.1104/pp.126.3.1232 11457973 PMC116479

[B69] Skopelitis DS Paranychianakis NV Paschalidis KA Pliakonis ED Delis ID Yakoumakis DI Kouvarakis A Papadakis AK Stephanou EG Roubelakis-Angelakis KA Abiotic stress generates ROS that signal expression of anionic glutamate dehydrogenases to form glutamate for proline synthesis in tobacco and grapevine Plant Cell 2006 18 10 2767 2781 10.1105/tpc.105.038323 17041150 PMC1626620

[B70] Chen WP Li PH Membrane stabilization by abscisic acid under cold aids proline in alleviating chilling injury in maize (Zea mays L.) cultured cells Plant Cell Environ 2002 25 8 955 962 10.1046/j.1365-3040.2002.00874.x

[B71] Pryor A A Maize Glutamic Dehydrogenase Null Mutant Is Cold Temperature Sensitive Maydica 1990 35 4 367 372

[B72] Hancock JT Henson D Nyirenda M Desikan R Harrison J Lewis M Hughes J Neill SJ Proteomic identification of glyceraldehyde 3-phosphate dehydrogenase as an inhibitory target of hydrogen peroxide in Arabidopsis Plant Physiol Bioch 2005 43 9 828 835 10.1016/j.plaphy.2005.07.012 16289945

[B73] Mateos RM Bonilla-Valverde D del Rio LA Palma JM Corpas FJ NADP-dehydrogenases from pepper fruits: effect of maturation Physiol Plant 2009 135 2 130 139 10.1111/j.1399-3054.2008.01179.x 19055545

[B74] Etienne C Moing A Dirlewanger E Raymond P Monet R Rothan C Isolation and characterization of six peach cDNAs encoding key proteins in organic acid metabolism and solute accumulation: involvement in regulating peach fruit acidity Physiol Plant 2002 114 2 259 270 10.1034/j.1399-3054.2002.1140212.x 11903973

[B75] Saito K Takagi Y Ling HC Takahashi H Noji M Molecular cloning, characterization and expression of cDNA encoding phosphoserine aminotransferase involved in phosphorylated pathway of serine biosynthesis from spinach Plant Mol Biol 1997 33 2 359 366 10.1023/A:1005730725764 9037153

[B76] Liepman AH Olsen LI Genomic analysis of aminotransferases in Arabidopsis thaliana Crit Rev Plant Sci 2004 23 1 73 89 10.1080/07352680490273419

[B77] Livio T Tadiello A Casadoro G Variations of the peach fruit transcriptome during ripening and in response to hormone treatments Caryologia 2007 60 1-2 156 159

[B78] Tadege M Dupuis II Kuhlemeier C Ethanolic fermentation: new functions for an old pathway Trends Plant Sci 1999 4 8 320 325 10.1016/S1360-1385(99)01450-8 10431222

[B79] Moyano E Encinas-Villarejo S Lopez-Raez JA Redondo-Nevado J Blanco-Portales R Bellido ML Sanz C Caballero JL Munoz-Blanco J Comparative study between two strawberry pyruvate decarboxylase genes along fruit development and ripening, post-harvest and stress conditions Plant Sci 2004 166 4 835 845 10.1016/j.plantsci.2003.09.025

[B80] Martin M Funk HT Serrot PH Poltnigg P Sabater B Functional characterization of the thylakoid Ndh complex phosphorylation by site-directed mutations in the ndhF gene Biochim Biophys Acta 2009 1787 7 920 928 10.1016/j.bbabio.2009.03.001 19272354

[B81] Zapata JM Guera A Esteban-Carrasco A Martin M Sabater B Chloroplasts regulate leaf senescence: delayed senescence in transgenic ndhF-defective tobacco Cell Death Differ 2005 12 10 1277 1284 10.1038/sj.cdd.4401657 15905880

[B82] Kahlau S Bock R Plastid transcriptomics and translatomics of tomato fruit development and chloroplast-to-chromoplast differentiation: chromoplast gene expression largely serves the production of a single protein Plant Cell 2008 20 4 856 874 10.1105/tpc.107.055202 18441214 PMC2390737

[B83] Gebbie LK Burn JE Hocart CH Williamson RE Genes encoding ADP-ribosylation factors in Arabidopsis thaliana L. Heyn.; genome analysis and antisense suppression J Exp Bot 2005 56 414 1079 1091 10.1093/jxb/eri099 15723828

[B84] Vernoud V Horton AC Yang Z Nielsen E Analysis of the small GTPase gene superfamily of Arabidopsis Plant Physiol 2003 131 3 1191 1208 10.1104/pp.013052 12644670 PMC166880

[B85] Matheson LA Hanton SL Rossi M Latijnhouwers M Stefano G Renna L Brandizzi F Multiple roles of ADP-ribosylation factor 1 in plant cells include spatially regulated recruitment of coatomer and elements of the Golgi matrix Plant Physiol 2007 143 4 1615 1627 10.1104/pp.106.094953 17307898 PMC1851833

[B86] Gonzalez-Aguero M Pavez L Ibanez F Pacheco I Campos-Vargas R Meisel LA Orellana A Retamales J Silva H Gonzalez M Identification of woolliness response genes in peach fruit after post-harvest treatments J Exp Bot 2008 59 8 1973 1986 10.1093/jxb/ern069 18453640 PMC2413281

[B87] Gerke V Creutz CE Moss SE Annexins: Linking Ca2+ signalling to membrane dynamics Nature Reviews Molecular Cell Biology 2005 6 6 449 461 10.1038/nrm1661 15928709

[B88] Monastyrskaya K Babiychuk EB Draeger A The annexins: spatial and temporal coordination of signaling events during cellular stress Cell Mol Life Sci 2009 66 16 2623 2642 10.1007/s00018-009-0027-1 19381436 PMC11115530

[B89] Reddy AM Reddy VS Scheffler BE Wienand U Reddy AR Novel transgenic rice overexpressing anthocyanidin synthase accumulates a mixture of flavonoids leading to an increased antioxidant potential Metab Eng 2007 9 1 95 111 10.1016/j.ymben.2006.09.003 17157544

[B90] Bianco A Cavarischia C Guiso M Total synthesis of anthocyanidins via Heck reaction Nat Prod Res 2006 20 1 93 97 10.1080/14786410500059474 16286317

[B91] Zhang ZQ Pang XQ Ji ZL Jiang YM Role of anthocyanin degradation in litchi pericarp browning Food Chemistry 2001 75 2 217 221 10.1016/S0308-8146(01)00202-3

[B92] Zhang ZQ Pang XQ Duan XW Ji ZL Jiang YM Role of peroxidase in anthocyanin degradation in litchi fruit pericarp Food Chemistry 2005 90 1-2 47 52 10.1016/j.foodchem.2004.03.023

[B93] Ogundiwin EA Peace CP Nicolet CM Rashbrook VK Gradziel TM Bliss FA Parfitt D Crisosto CH Leucoanthocyanidin dioxygenase gene (PpLDOX): a potential functional marker for cold storage browning in peach Tree Genet Genomes 2008 4 3 543 554 10.1007/s11295-007-0130-0

[B94] Lo Piero AR Mercurio V Puglisi I Petrone G Gene isolation and expression analysis of two distinct sweet orange [Citrus sinensis L. (Osbeck)] tau-type glutathione transferases Gene 2009 443 1-2 143 150 10.1016/j.gene.2009.04.025 19422890

[B95] Vizoso P Meisel LA Tittarelli A Latorre M Saba J Caroca R Maldonado J Cambiazo V Campos-Vargas R Gonzalez M Comparative EST transcript profiling of peach fruits under different post-harvest conditions reveals candidate genes associated with peach fruit quality BMC Genomics 2009 10 1 423 10.1186/1471-2164-10-423 19744325 PMC2748099

[B96] Obenland DM Vensel WH Hurkman WJ Alterations in protein expression associated with the development of mealiness in peaches Journal of Horticultural Science & Biotechnology 2008 83 1 85 93

[B97] Abdi N Holford P Mcglasson B Application of two-dimensional gel electrophoresis to detect proteins associated with harvest maturity in stonefruit Postharvest Biol Tec 2002 26 1 1 13 10.1016/S0925-5214(01)00197-1

[B98] Bradford MM A rapid and sensitive method for the quantitation of microgram quantities of protein utilizing the principle of protein-dye binding Anal Biochem 1976 72 248 254 10.1016/0003-2697(76)90527-3 942051

[B99] Durbin BP Hardin JS Hawkins DM Rocke DM A variance-stabilizing transformation for gene-expression microarray data Bioinformatics 2002 18 Suppl 1 S105 110 12169537 10.1093/bioinformatics/18.suppl_1.s105

[B100] Huber W von Heydebreck A Sultmann H Poustka A Vingron M Variance stabilization applied to microarray data calibration and to the quantification of differential expression Bioinformatics 2002 18 Suppl 1 S96 104 12169536 10.1093/bioinformatics/18.suppl_1.s96

[B101] Kreil DP Karp NA Lilley KS DNA microarray normalization methods can remove bias from differential protein expression analysis of 2D difference gel electrophoresis results Bioinformatics 2004 20 13 2026 2034 10.1093/bioinformatics/bth193 15044229

[B102] Urfer W Grzegorczyk M Jung K Statistics for proteomics: A review of tools for analyzing experimental data Proteomics 2006 48 55 10.1002/pmic.200600554 17031797

[B103] Latorre M Silva H Saba J Guziolowski C Vizoso P Martinez V Maldonado J Morales A Caroca R Cambiazo V JUICE: a data management system that facilitates the analysis of large volumes of information in an EST project workflow Bmc Bioinformatics 2006 7 10.1186/1471-2105-7-513 17123449 PMC1676024

