# Supplementary figures and images for: Proteomic analysis of peach fruit mesocarp softening and chilling injury using difference gel electrophoresis (DIGE)
Source: BMC Genomics. 2010 Jan 18;11:43. doi: 10.1186/1471-2164-11-43 (PMC2822761; doi:10.1186/1471-2164-11-43)

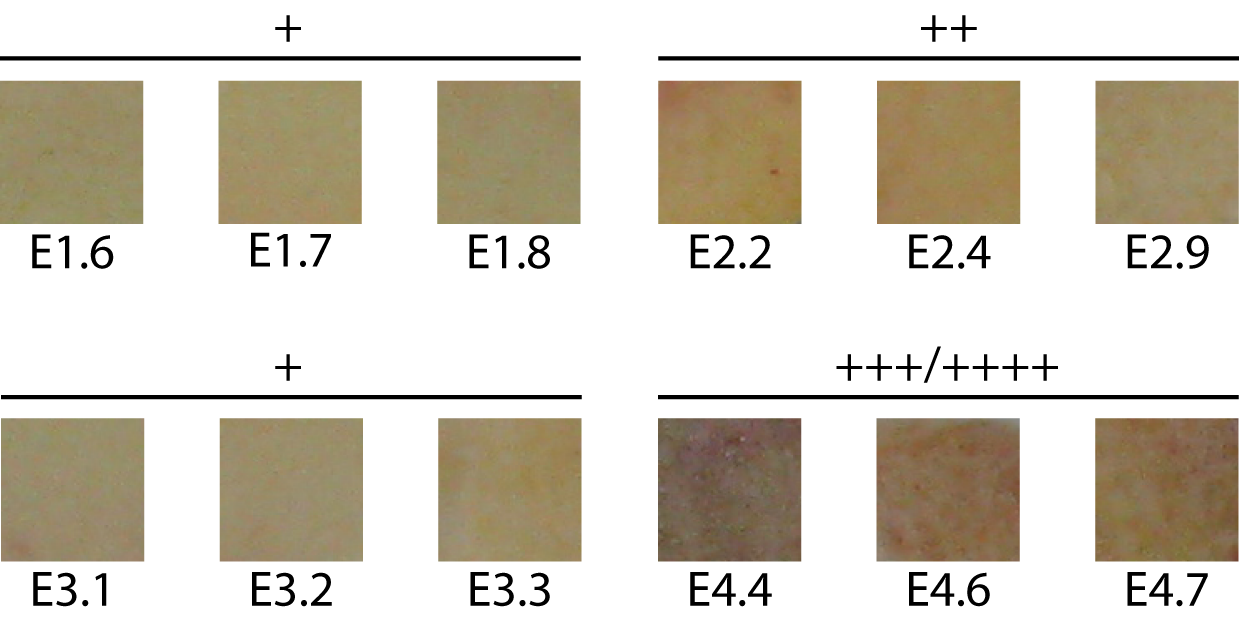

Supplement: Additional file 1 — Evaluation of the mesocarp browning degree among fruits from the four different postharvest conditions evaluated. Fruit mesocarp images from three representative samples from the E1, E2, E3 and E4 conditions are shown. The degree of browning was assessed visually and values were assigned to each condition using E1 as standard. As can be seem, the fruits from the E4 condition have a darker brown mesocarp color when compared to the other four conditions. + no browning; ++ no browning degree with a color change of the mesocarp; +++ medium browning degree; ++++ high browning degree. [file 1471-2164-11-43-S1.TIFF]
