# Supplementary material for: Proteomic analysis of peach fruit mesocarp softening and chilling injury using difference gel electrophoresis (DIGE)
Source: BMC Genomics. 2010 Jan 18;11:43. doi: 10.1186/1471-2164-11-43 (PMC2822761; doi:10.1186/1471-2164-11-43)
Supplement: Additional file 2 — Peptide sequences obtained by mass spectrometry and their localization within proteins. A compendium of the protein sequences containing the peptide sequences (highlighted) delivered by the mass spectra is presented. [file 1471-2164-11-43-S2.PDF]

Additional file 2

>Low molecular weight heat shock protein [Malus x domestica]; N004
MSLIPNSRRGSSSVFDPFSLNLWDPFKDFPFPSSSSLSAFPEFSRENSAFVNTRVDWKETPEAHVFKADVPG
LKKEEVKVEVEDDRVLKISGERNVEEEDKNDKWYRVERSSGKFLRRFQLPENAKVDQIKAAMENGVLSVTVP
KAELKNVDVRAIEISG

>Glyceraldehyde-3-phosphate dehydrogenase [Nicotiana tabacum]; N005
MAKVKIGINGFGRIGRLVARVALQRDDVELVAVNDPFISVEYMTYMFKYDSVHGQWKHHELKVKDDKTLLFG
EKAVTVFGFRNPEEIPWGQTGADYIVESTGVFTDKDKAAAHLKGGAKKVIISAPSKDAPMFVVGVNEKEYKP
ELNIVSNASCTTNCLAPLAKVINDRFGIVEGLMTTVHSITATQKTVDGPSAKDWRGGRAASFNIIPSSTGAA
KAVGKVLPALNGKLTGMAFRVPTVDVSVVDLTVRLEKEATYDEIKAAIKEESEGKLKGILGYTEDDVVSTDF
VGDNRSSIFDAKAGIALSKNFVKLVSWYDNEWGYSTRVVDLIKHMASVQ

>Low molecular weight heat shock protein [Malus x domestica]; N011
MSLIPNSRRGSSSVFDPFSLNLWDPFKDFPFPSSSSLSAFPEFSRENSAFVNTRVDWKETPEAHVFKADVPG
LKKEEVKVEVEDDRVLKISGERNVEEEDKNDKWYRVERSSGKFLRRFQLPENAKVDQIKAAMENGVLSVTVP
KAELKNVDVRAIEISG

>ADP-ribosylation factor B1C [Arabidopsis thaliana]; N012
MGQTFRKLFDTFFGNQEMRVVMLGLDAAGKTTILYKLHIGEVLSTVPTIGFNVEKVQYKNVIFTVWDVGGQE
KLRPLWRHYFNNTDGLIYVVDSLDRERIGKAKQEFQDIIRDPFMLNSVILVFANKQDMRGAMSPREVCEGLG
LLDLKNRKWHIQGTCALQGDGLYEGLDWLSATLKEVKAAGYSSVGPSF

>ADP-ribosylation factor 1 [Solanum tuberosum]; N014
MGLTISKLFSRLFAKKEMRILMVGLDAAGKTTILYKLKLGEIVTTIPTIGFNVETVEYKNISFTVWDVGGQD
KIRPLWRHYFQNTQGLIFVVDSNDRDRVNEAREELMRMLAEDELRDAVLLVFANKQDLPNAMNAAEITDKLG
LHSLRQRHWYIQSTCATSGEGLYEGLDWLSNQIRNQKANCNGTMLLWLLYPEK

>PRU1_PRUAV Major allergen Pru av 1 (Pru a 1) [Prunus avium]; N021
MGVFTYESEFTSEIPPPRLFKAFVLDADNLVPKIAPQAIKHSEILEGDGGPGTIKKITFGEGSQYGYVKHKI
DSIDKENYSYSYTLIEGDALGDTLEKISYETKLVASPSGGSIIKSTSHYHTKGNVEIKEEHVKAGKEKASNL
FKLIETYLKGHPDAYN

>Small heat shock protein [Retama raetam]; N023
MSLIPSVFGTGRRTNVFDPFSLDIWDPFQDFPLRTIAPSGFDTETAAVANTRIDWKETPEAHVFKADLPGLK
KEEVKVEVEEGRVLQISGERTKEKEDKNDTWHRVECSAGRFLRRFRLPENAKVEQVKASLENGVLTVTVPKE
EVKKPDVKPVQITG

>Peroxiredoxin [Populus tremula x Populus tremuloides]; N026
MAPIAVGDVLPDGKLAYFDEQDQLQEVSVHSLVAGKKVILFGVPGAFTPTCSLKHVPGFIEKAGELKSKGVT
EILCISVNDPFVMKAWAKSYPENKHVKFLADGSATYTHALGLELDLQEKGLGTRSRRFALLVDDLKVKAANI
EGGGEFTVSSAEDILKDL

>Low molecular weight heat shock protein [Malus x domestica]; N027
MSLIPNSRRGSSSVFDPFSLNLWDPFKDFPFPSSSSLSAFPEFSRENSAFVNTRVDWKETPEAHVFKADVPG
LKKEEVKVEVEDDRVLKISGERNVEEEDKNDKWYRVERSSGKFLRRFQLPENAKVDQIKAAMENGVLSVTVP
KAELKNVDVRAIEISG

>Eukaryotic translation initiation factor 4D [Medicago sativa]; N028
MSDEEHQFESKADAGASKTYPQQAGTIRKNGYIVIKNRPCKVVEVSTSKTGKHGHAKCHFVAIDIFTSKKLE
EVYVPSSHNCDVPHVNRTDYQLIDISEDGFVSLLTENGNTKDDLKLPTDDSLLTQIKDGFAEGKDLVVSVMS
AMGEEQICALKDIGGKN

>Major allergen Pru p 1 [Prunus persica]; N029
MPLCSLFILLILNLLIITMGVFTYESEFTSEIPPPRLFKAFVLDADNLVPKIAPQAIKHSEILEGDGGPGTI
KKITFGEGSQYGYVKHKIDSIDKENHSYSYTLIEGDALGDNLEKISYETKLVASPSGGSIIKSTSHYHTKGD
VEIKEEHVKAGKEKASNLFKLIETYLKGHPDAYN

Additional file 2 (*continuation*)

>Small heat shock protein [Prunus persica]; N031
MALSLFGGRRSNVFDPFSLDIWDPLEGLGTLANIPPSARETTAIANTRIDWKETPEAHIFIADLPGLKKEEV
KVEVDDGKVLHISGERSREQEEKNDKWHRIERSTGKFSRRFRLPDNAKIDQVKASMENGVLTVTVPKEEEKR
PQVKAIDISG

>Small heat shock protein [Prunus persica]; N032
MALSLFGGRRSNVFDPFSLDIWDPLEGLGTLANIPPSARETTAIANTRIDWKETPEAHIFIADLPGLKKEEV
KVEVDDGKVLHISGERSREQEEKNDKWHRIERSTGKFSRRFRLPDNAKIDQVKASMENGVLTVTVPKEEEKR
PQVKAIDISG

>Superoxide dismutase [Nicotiana plumbaginifolia]; N033
MVKAVAVLSSSEGVSGTIFFTQDGDAPTTVTGNVSGLKPGLHGFHVHALGDTTNGCMSTGPHYNPAGKEHGA
PEDEVRHAGDLGNITVGEDGTASFTLTDKQIPLAGPQSIIGRAVVVHADPDDLGKGGHELSKTTGNAGGRVA
CGIIGLQG

>Thioredoxin H [Prunus persica]; N035
MAEENQVIGCHTTQAWEEQLHKGNENKKLVVVDFTASWCGPCRLIAPILAELAKKTPEVTFLKVDVDELRTV
SEEWGVEAMPTFLFLKEGKIVDKVVGAKKDELQIKVAKHVAAAAAASATSASATAATATATASA

>Low molecular weight heat shock protein [Malus x domestica]; N037
MSLIPNSRRGSSSVFDPFSLNLWDPFKDFPFPSSSSLSAFPEFSRENSAFVNTRVDWKETPEAHVFKADVPG
LKKEEVKVEVEDDRVLKISGERNVEEEDKNDKWYRVERSSGKFLRRFQLPENAKVDQIKAAMENGVLSVTVP
KAELKNVDVRAIEISG

>NADH dehydrogenase subunit F [Graffenrieda latifolia]; N042
LFLGSGSIIHSMEVIVGYSPDKSQNIVLMGGLKKHVPITKTAFLIGTLSLCGIPPLACFWSKDEILNDSWLY
SPVFAIIACFTAGLTAFYMFRIYLLTFEGHLNVHFQNYSGQKSGSCYSISPWGKEIQKNKFSLLSTTINEKG
ILFFFNTTYRIFDNEKKMIRPFISIPYFGIQNTFFYPPESDSTMLFSMSILVLFTCLVGIIGIPYNQSGIYF
DLLSNLLNPSINLLHPNQNNSRDWYEFIINALFSVSLA

>Manganese superoxide dismutase 1 [Prunus persica]; N053
MALRTLLTRKALTGSGLGLGFQSIRGFQTFSLPDLPYDYSALEPAISGEIMQLHHQKHHKTYVTNYNKALEQ
LDDAMAKGDAPTTVKLQSAIKFNGGGHINHSLFWQNLAPVREDGGEPPKGSLGWAIDTNFGSLEALIQKMNA
EGAALRGSGWVWLGVDKELKKLVVETTANQDPLVTKGPSLVPLLGLDVWEHAYYLQYKNVRPDYLKNIWKVV
SWKYASEVYEKVCP

>Putative quinone reductase [Vitis vinifera]; N056
MATKVYIVYYSMYGHVEKLAEEIRKGAASVEGVEAKLWQVPETLHEEALSKMSAPPKSESPIITPNELAEAD
GFVFGFPTRFGMMAAQFKAFLDATGGLWRAQALAGKPAGIFYSTGSQGGGQETTALTAITQLVHHGMLFVPI
GYTFGAGMFEMENVKGGSPYGAGTFAGDGSRQPTELELQQAFHQGQYIASITKKLKGSTA

>Pectin methylesterase [Nicotiana tabacum]; N073
KRYVIRIKAGVYRENVDVPKKKTNIMFMGDGRSNTIITGSRNVKDGSTTFNSATVAAVGEKFLARDITFQNT
AGAAKHQAVALRVGSDLSAFYRRDILAYQDSLYVHSNRQYFVQCLIAGTVDFIFGNAAAVLQNCDIHARRPG
SGQKNMVTAQGRTDPNQNTGVVIQNCRIGATSDLRPVQKSFPTYLGRPWKEYSRTVIMQSSITDVINSAGWH
EWNGNFALNTLFYGEYQNTGAGAGTSGRVKWRGFKVITSATEAQAYTPGR

Additional file 2 (*continuation*)

>Porin [Prunus armeniaca]; N077
MVKGPGLYLDIGKKARDLLYKDYQSDHKFTVTTYTSTGVAISSTGIRKGDLYLGDVSTQLKNKNITTDVKVD
TDSNLRTTITIDEPAPGLKAIFSFIVPDQRSGKVELQYQHEYAGISTSIGLTANPIVNFSGVVGNNLLSLGT
DLSFDTASGNFTKCNAGLNFTHTDLIASLILNDKADTVTASYYHTVSPLTNTAVGAELSHSFSSNENSLTIG
TQHALDPLTTVKGRVNNYGRASALIQHEWRPKSFFTISGEVDTRAIEKSAKIGLALALKP

>Triose phosphate isomerase cytosolic isoform [Solanum chacoense]; N083
MGRTFFVGGNWKCNGTSEEIKKIVATLNAGQVPSQDVVEVVVSPPYVFLPLVKNELRSDFHVAAQNCWVKKG
GAFTGEVSADMLVNLGIPWVILGHSERRAILGESNEFVGDKVAYALSQGLRVIACVGETLEQRESGSTMDVV
AAQTKAIAERVKDWSNVVVAYEPVWAIGTGKVATPAQAQEVHAELRKWLQANVSAEVAASTRIIYGGSVSGA
NCKELAGQPDVDGFLVGGASLKPEFIDIIKAAEVKKGA

>Proteasome subunit alpha type 7 [Cicer arietinum]; N084
MARYDRAITVFSPDGHLFQVEYALEAVRKGNAAVGVRGTDNVVLGVEKKSTAKLQDTRSVRKIVNLDDHIAL
ACAGLKADARVLINRARVECQSHRLTVEDPVTVEYITRYIAGLQQKYTQSGGVRPFGLSTLIVGFDPYTGSP
SLYQTDPSGTFSAWKANATGRNSNSIREFLEKNFKETSGQETVKLAIRALLEVVESGGKNIEVAVMTKENGL
RQLEEAEIDAIVAEIEAEKAAAEAAKKAPPKDT

>Cytosolic ascorbate peroxidase [Fragaria x ananassa]; N086
MGKCYPTVSEEYKKAIDKAKRKLRGLIAEKNCAPLMLRLAWHSAGTYDVKTKTGGPFGTMKQPAELAHGANN
GLDIAVRLLEPIKEQFPILSYADFYQLAGVVAVEVTGGPDVPFHPGREDKPEPPPEGRLPDAGKGSDHLREV
FGKTMGLSDQDIVALSGGHTLGRAHKERSGFEGPWTPNPLIFDNSYFTVLLSGEKEGLLQLPTDKALLSDPV
FRPLVEKYAADEDAFFADYALAHQRLSELGFAEA

>Abscisic stress ripening-like protein [Prunus persica]; N093
MSEEKHHHGLFHHHKDEDRPIETSDYPQSGGYSDEGRTGSGYGGGGGYGDGGGGYGDNTAYSGEGRPGSGYG
GGGGYGESADYSDGGRYKETAAYGTTGTHESEIDYKKEEKHHKHLEHLSEAGAAAAGVFALHEKHESKKDPE
HAHRHKIEEEIAAAAAVGSGGFAFHEHHEKKEAKEEEEESHGKKHHHLF

>Quinone-oxidoreductase QR2 [Triphysaria versicolor]; N096
MATKVYIVYYSTYGHVERLAQEIKKGAESVGNVEVKLWQVPEILSDEVLGKMWAPPKSDVPVITPDELVEAD
GIIFGFPTRFGMMAAQFKAFFDSTGGLWKTQALAGKPAGIFFSTGTQGGGQETTALTAITQLTHHGMIYVPI
GYTFGADMFNMEKIKGGSPYGAGTFAGADGSRQPSDIELKQAFHQGMYIAGITKKIKQTSA

>Iron-binding protein [Pyrus pyrifolia]: N111
MSTTSLRAISTFSVPSKLGDQGGAVSTLLSNSKLGSSSSALSFKPQRKLEKFAVSASSEAVALTGVVFQPFE
EVKNDAFVVPVSPQVSLARQRYTDESEAATNEQINVEYNVSYVYHALFAYFDRDNVALKGLAKFFKESSEEE
REHAEKLMEYQNKRGGRVKLHSVIAAPTEFDHAEKGDALYAMELALSLEKLTNEKLLNLHKVADQNNDPQLM
DFIESEFLAEQVEAIKKIADYVTQLRRVGKGHGVWHFDQYLLHEGDAAN

>Annexin [Medicago sativa]; N138
SHVPSPSEDSEQLRGAFQGWGTNEGLIISILAHRNAAQRKSIRETYTQTHGEDLLKDLDKELSSDFEKAVLL
WTLDPAERDAFLANQATKMLTSNNSIIVEIASTRSPLELLKAKQAYQVRFKKSLEEDVAYHTSGDIRKLLVP
LVGIHRYEGDEVNMTLAKSEAKLLHEKIADKAYNHDDLIRIVTTRSKAQLNATLNHYNNEFGNVIDKDLETD
SDDEYLKLLRAAIKGLTYPEKYFEELLRLAINKMGTDENALTRVVTTRAEVDLQRIAEEYQRRNSVPLDRAI
DKDTSGDYQKILLALMGHDE

>Annexin [Medicago sativa]; N139
SHVPSPSEDSEQLRGAFQGWGTNEGLIISILAHRNAAQRKSIRETYTQTHGEDLLKDLDKELSSDFEKAVLL
WTLDPAERDAFLANQATKMLTSNNSIIVEIASTRSPLELLKAKQAYQVRFKKSLEEDVAYHTSGDIRKLLVP
LVGIHRYEGDEVNMTLAKSEAKLLHEKIADKAYNHDDLIRIVTTRSKAQLNATLNHYNNEFGNVIDKDLETD
SDDEYLKLLRAAIKGLTYPEKYFEELLRLAINKMGTDENALTRVVTTRAEVDLQRIAEEYQRRNSVPLDRAI
DKDTSGDYQKILLALMGHDE

Additional file 2 (*continuation*)

>NAD-dependent malate dehydrogenase [Prunus persica]; N140
MAKGPVRVLVTGAAGQIGYALVPMIARGVMLGADQPVILHLLDIPPAAEALNGVKMELVDAAFPLLKGVVAT
TDVVEACTGVNIAVMVGGFPRKEGMERKDVMSKNVSIYKSQASALEKHAAANCKVLVVANPANTNALILKEF
APSIPEKNITCLTRLDHNRALGQVSERLNVQVSDVKNVIIWGNHSSSQYPDVNHATVKTPSGEKAVRELVAD
DAWLTGEFITTVQQRGAAIIKARKLSRALSAASSACDHIRDWVLGTPEGTWVSMGVYSDGSYNVPSGLIYSF
PVTCQNGEWKIVQGLSIDEFSRKKLDATADELSEEKALAYSCLS

>Cytosolic aldolase [Fragaria x ananassa]; N155
MTAYRGKYADELIKNAAYIGTPGKGILAADESTGTIGKRFDSIKVENVEENRRALRELLFTAPGVLQYLSGV
ILFEETLYQKTAEGKPFVDVLNEGGVLPGIKVDKGVVELKGTDGETTTQGLDGLGARCAKYYEAGARFAKWR
AVLKIGPNEPSQLSINENANGLARYAIICQENGLVPIVEPEILVDGPHSIDKCADVTERVLAACYKALNDHH
VLLEGTLLKPNMVTPGSEAKKASPEVIAEYTVRALQRTTPAAVPAVVFLSGGQSEEEATLNLNAMNQLKGKK
PWTLSFSFGRALQASTLKAWSGKKENVKAAQEALLTRAKANSEATLGTYKGDAKLGEGAAESLHVKDYKY

>1-aminocyclopropane-1-carboxylate oxidase [Prunus persica]; N162
MENFPIINLEGLNGEGRKATMEKIKDACENWGFFELVSHGIPTEFLDTVERLTKEHYRQCLEQRFKELVASK
GLEAVKTEVNDMDWESTFYLRHLPKSNISEVPDLEDQYRNVMKEFALKLEKLAEQLLDLLCENLGLEQGYLK
KAFYGTNGPTFGTKVSNYPPCPNPELIKGLRAHTDAGGLILLFQDDKVSGLQLLKDGQWIDVPPMRHSIVIN
LGDQLEVITNGKYKSVEHRVIAQTDGTRMSIASFYNPGSDAVIYPAPTLVEKEAEEKNQVYPKFVFEDYMKL
YAGLKFQPKEPRFEAMKAVETNISLGPIATA

>1-aminocyclopropane-1-carboxylate oxidase [Prunus persica]; N163
MENFPIINLEGLNGEGRKATMEKIKDACENWGFFELVSHGIPTEFLDTVERLTKEHYRQCLEQRFKELVASK
GLEAVKTEVNDMDWESTFYLRHLPKSNISEVPDLEDQYRNVMKEFALKLEKLAEQLLDLLCENLGLEQGYLK
KAFYGTNGPTFGTKVSNYPPCPNPELIKGLRAHTDAGGLILLFQDDKVSGLQLLKDGQWIDVPPMRHSIVIN
LGDQLEVITNGKYKSVEHRVIAQTDGTRMSIASFYNPGSDAVIYPAPTLVEKEAEEKNQVYPKFVFEDYMKL
YAGLKFQPKEPRFEAMKAVETNISLGPIATA

>1-aminocyclopropane-1-carboxylate oxidase [Prunus persica]; N164
MENFPIINLEGLNGEGRKATMEKIKDACENWGFFELVSHGIPTEFLDTVERLTKEHYRQCLEQRFKELVASK
GLEAVKTEVNDMDWESTFYLRHLPKSNISEVPDLEDQYRNVMKEFALKLEKLAEQLLDLLCENLGLEQGYLK
KAFYGTNGPTFGTKVSNYPPCPNPELIKGLRAHTDAGGLILLFQDDKVSGLQLLKDGQWIDVPPMRHSIVIN
LGDQLEVITNGKYKSVEHRVIAQTDGTRMSIASFYNPGSDAVIYPAPTLVEKEAEEKNQVYPKFVFEDYMKL
YAGLKFQPKEPRFEAMKAVETNISLGPIATA

>Oxidoreductase [Arabidopsis thaliana]; N165
MKAWVYSDYGGVDVLKLESNIVVPEIKEDQVLIKVVAAALNPVDAKRRQGKFKATDSPLPTVPGYDVAGVVV
KVGSAVKDLKEGDEVYANVSEKALEGPKQFGSLAEYTAVEEKLLALKPKNIDFAQAAGLPLAIETADEGLVR
TEFSAGKSILVLNGAGGVGSLVIQLAKHVYGASKVAATASTEKLELVRSLGADLAIDYTKENIEDLPDKYDV
VFDAIGMCDKAVKVIKEGGKVVALTGAVTPPGFRFVVTSNGDVLKKLNPYIESGKVKPVVDPKGPFPFSRVA
DAFSYLETNHATGKVVVYPIP

>Actin [Helianthus annuus]; N170
MAHEGEIQPLVCDNGTGMVKAGFAGDDAPRAVFPSIVGRPRHTGVMVGMGQKDAYVGDEAQSKRGILTLKYP
IEHGIVSNWDDMEKIWHHTFYNELRVAPEEHPVLLTEAPLNPKANREKMTQIMFETFNVPAMYVAIQAVLSL
YASGRTTGIVLDSGDGVSHTVPIYKGYALPHAILRLDLAGRDLTDSLMKILTERGYMFTTTAEREIVRDMKE
KLAYVALDYEQELETAKSSSSVEKNYELPDGQVITIGAERFRCPEVLFQPSLIGMEAAGIHETTYNSIMKCD
VDIRKDLYGNIVLSGGSTMFPGIADRMSKEITALAPSSMKIKVVAPPERKYSVWIGGSILASLSTFQQMWIS
KGEYDESGPSIVHRKCF

Additional file 2 (*continuation*)

>Actin [Helianthus annuus]; N172
MADAEDIQPLVCDNGTGMVKAGFAGDDAPRAVFPSIVGRPRHTGVMVGMGQKDAYVGDEAQSKRGILTLKYP
IEHGIVSNWDDMEKIWHHTFYNELRVAPEEHPVLLTEAPLNPKANREKMTQIMFETFNVPAMYVAIQAVLSL
YASGRTTGIVLDSGDGVSHTVPIYEGYALPHAILRLDLAGRDLTDALMKILTERGYSFTTTAEREIVRDVKE
KLAYVALDYEQELETAKSSSSIDKSYELPDGQVITIGAERFRCPEVLFQPSLIGMEAAGIHETTYNSIMKCD
VDIRKDLYGNIVLSGGSTMFPGIADRMSKEITALAPSSMKIKVVAPPERKYSVWIGGSILASLSTFQQMWIA
KAEYDESGPSIVHRKCF

>Alpha-1,4-glucan-protein synthase [UDP-forming]; N183
MASLPKPTPLLKDELDIVIPTIRNLDFLEMWRPFFEQYHLIIVQDGDPSKVIKVPEGFDYELYNRNDINRIL
GPKASCISFKDSACRCFGYMVSKKKYIYTIDDDCFVAKDPTGHEINALEQHIKNLLSPSTPFFFNTLYDPYR
EGTDFVRGYPFSLREGVPTAVSHGLWLNIPDYDAPTQLVKPHERNTRFVDAVLTIPKGSLFPMCGMNLAFNR
ELIGPAMYFGLMGDGQPIGRYDDMWAGWCIKVICDHLGYGVKTGLPYIWHSKASNPFVNLKKEYKGIFWQEE
IIPFFQAATLSKDCTSVQKCYIELSKQVKEKLGTIDPYFIKLADAMVTWVEAWDEINNNKSEETTSTKASEV
AATK

>Anthocyanidin synthase [Prunus persica]; N187
DEGPQVPTIDLKEIDSENENVRERCREELKKAAVDWGVMHLVNHGISDELMDRVRKAGKAFFDLPIEQKEKY
ANDQASGKIQGYGSKLANNASGQLEWEDYFFHLVYPEDKRDLSIWPQTPADYIEATAEYAKELRALATKVLR
VLSLGLGLEEGRLEKEVGGLEELLLQMKINYYPLCPQPELALGVEAHTDVSALTFILHNMVPGLQLFYEGKW
VTAKCVPNSIIMHIGDTIEILSNGKYKSILHRGMVNKEKVRISWAVFCEPPKEKIILK

>Endopolygalacturonase [Prunus persica]; N193
MANRRSLFSLSLIFVFMINSAIATPVTYNVASLGAKADGKTDSTKAFLSAWAKACASMNPGVIYVPAGTFFL
RDVVFSGPCKNNAITFRIAGTLVAPSDYRVIGNAANWIFFHHVNGVTISGGILDGQGTALWACKASHGESCP
SGATTLGFSDSNNIVVSGLASLNSQMFHIVINDCQNVQMQGVRVSASGNSPNTDGIHVQMSSGVTILNSKIA
TGDDCVSIGPGTSNLWIEGVACGPGHGISIGSLGKEQEEAGVQNVTVKTVTFTGTQNGLRIKSWGRPSTGFA
RNILFQHATMVNVENPIVIDQHYCPDNKGCPGQVSGVQISDVTYEDIHGTSATEVAVKFDCSPKHPCSEIKL
EDVKLTYKNQAAESSCSHADGTTEGVVQPTSCL

>NADP-dependent isocitrate dehydrogenase [Prunus persica]; N201
MAFQKIKVANPIVEMDGDEMTRVFWKSIKDKLILPFLELDIKYFDLGLPHRDATDDKVTVESAEATLKYNVA
IKCATITPDEGRVKEFNLKSMWRSPNGTIRNILNGTVFREPILCKNIPRLIPGWTKPICIGRHAFGDQYRAT
DAVIKGPGKLKLVFVPDGKDEKTELEVYNFTGEGGVALAMYNTDESIRAFAEASMTTAYEKKWPLYLSTKNT
ILKKYDGRFKDIFQEVYEAKWKSQYEAAGIWYEHRLIDDMVAYALKSDGGYVWACKNYDGDVQSDFLAQGFG
SLGLMTSVLVCPDGKTIEAEAAHGTVTRHYRVHQKGGETSTNSIASIFAWTRGLAHRAKLDDNAKLLDFTQK
LEEACIGTVESGKMTKDLALIIHGPKLARNHYLNTEEFIEAVAEELRARLSLKE

>Glutamate Dehydrogenase 1; oxidoreductase [Arabidopsis thaliana]; N208
MNALAATNRNFKLAARLLGLDSKLEKSLLIPFREIKVECTIPKDDGTLASFVGFRVQHDNARGPMKGGIRYH
PEVDPDEVNALAQLMTWKTAVAKIPYGGAKGGIGCDPSKLSISELERLTRVFTQKIHDLIGIHTDVPAPDMG
TGPQTMAWILDEYSKFHGYSPAVVTGKPIDLGGSLGRDAATGRGVMFGTEALLNEHGKTISGQRFVIQGFGN
VGSWAAKLISEKGGKIVAVSDITGAIKNKDGIDIPALLKHTKEHRGVKGFDGADPIDPNSILVEDCDILVPA
ALGGVINRENANEIKAKFIIEAANHPTDPDADEILSKKGVVILPDIYANSGGVTVSYFEWVQNIQGFMWEEE
KVNDELKTYMTRSFKDLKEMCKTHSCDLRMGAFTLGVNRVAQATILRGWGA

>Phosphoserine aminotransferase, chloroplast precursor [Spinacia oleracea]; N209
MAMAATSSTQTNLFLKAPFNPQQNCQQTHFLPLNFKIRNPISRITCSATPTATAVSTTTKIDQRSEERVFNF
AAGPAVLPENVLQKAQSELLNWRGSGMSVMEMSHRGKEFTSIIDKAEADLRTLLNIPSDYTVLFLQGGASTQ
FSAIPLNLCTPDSAVDYIVTGSWGDKAAKEAAKYAAVSSIWSGKSDNYVRIPNFDGSEFVQNSQARYLHICA
NETIYGVEFKKYPVPANPDGFLVADMSSNFCSKPVDVTKFGLIYAGAQKNVGPSGVTIVIVRNDLIGNAQKM
TPVMLDYKIHADNKSLYNTPPCYGIYMCGLVFEDLLNQGGLVEVEKKNKAKAQVLYDAIDESNGFYKCPVEK
SVRSLMNVPFTLEKSELEGDFIKEAAKEKMVALKGHRSVGGMRASIYNAMPLAGVEKLVAFMKEFQAKHA

Additional file 2 (*continuation*)

>Quinone-oxidoreductase QR1 [Triphysaria versicolor]; N222
MAGKLMRAVQYDGYGGGAAGLKHVEVPIPSPGKGEVLIKLEAISLNQLDWKLQNGMVRPFLPRKFPFIPATD
VAGEVVRIGQDVKNFKPGDKVVAMLGSFGGGGLAEYGVASEKLTVHRPPEVSAAESSGLPIAGLTAHMALTQ
HIGLNLDKSGPHKNILITAASGGVGQYAVQLAKLGNTHVTATCGSRNFDLVKSLGADEVIDYKTPEGAALKS
PSGKKYDAVIHCASPLPWSVFKPNLSKHGKVIDITPGPRVMLTSAMTKLTCSKKRLVTLLVVIKGEHLSYLV
ELMREGKLKTVIDSKFSLSKAEEAWAKSIDGHATGKIVVEP

>Catalase [Prunus persica]; N228
MDPYKHRPSSAFDSPYWTTNAGAPVWNNDSSLTVGPRGPVLLEDYHLVEKLATFDRERIPERVVHARGASAK
GFFEVTHDISQLTCADFLRAPGVQTPVIVRFSTVIHERGSPETLRDPRGFAVKFYTREGNFDLVGNNFPVFF
VRDAMKFPDAIRAFKPNPKSHIQETWRILDFFSHLPESLHTFAFFYDDLGVPQDYRHMEGSSVHAYTLISKA
GKVHYVKFHWKPTCGVKCLLEDEAIKVGGANHSHATKDLYDSIAAGNYPEWKLYIQTMDPDHEDRFDFDPLD
LTKTWPEDILPLQPVGRLVLNKNIDNFFAENEQLAFNPAHVVPGIYYSDDKVLQTRIFAYSDTQRHRLGPNY
LQLPVNAPKCPHHNNHHEGFMNFMHRDEEVNYFPSRHDPVRHAERYPIPSNILSGKREKCVIEKENNFKQPG
ERYRSWAPDRQERFIRRWVDALSDPRVTHEIRSIWISYWSQADKSLGQKLLSRLNVRPSI

>Catalase [Prunus persica]; N229
MDPYKHRPSSAFDSPYWTTNAGAPVWNNDSSLTVGPRGPVLLEDYHLVEKLATFDRERIPERVVHARGASAK
GFFEVTHDISQLTCADFLRAPGVQTPVIVRFSTVIHERGSPETLRDPRGFAVKFYTREGNFDLVGNNFPVFF
VRDAMKFPDAIRAFKPNPKSHIQETWRILDFFSHLPESLHTFAFFYDDLGVPQDYRHMEGSSVHAYTLISKA
GKVHYVKFHWKPTCGVKCLLEDEAIKVGGANHSHATKDLYDSIAAGNYPEWKLYIQTMDPDHEDRFDFDPLD
LTKTWPEDILPLQPVGRLVLNKNIDNFFAENEQLAFNPAHVVPGIYYSDDKVLQTRIFAYSDTQRHRLGPNY
LQLPVNAPKCPHHNNHHEGFMNFMHRDEEVNYFPSRHDPVRHAERYPIPSNILSGKREKCVIEKENNFKQPG
ERYRSWAPDRQERFIRRWVDALSDPRVTHEIRSIWISYWSQADKSLGQKLLSRLNVRPSI

>Dehydrin-like protein [Prunus persica]; N231
KTDEYGNPVHHTTTGTGRTDEFGNPVQHGVADTGYGTGAGYGIHTKPGVVEHHGVPAVLHSKDDQYSRDTQT
TTGGYGGDGYTGGEHQEKKGLLGQLQDKLPGGNKDGQYSHDTQTTTGAYGGAGYTGGEHHEKKGVIGQVKDK
LPGGQKDDQYCRDTHPTTGAYGGAGYTGGEDHEKKGIIGQVKDKLPGGQKDDQYCRDTHPTTGAYGGAGYTG
GEHQEKKGIIGQVKDKLPGGQKDDQYCHDTHPTTGAYGGAGYTGGDTREKKGIIGQVKDKLPGGQKDDHYSH
DTHPTTGAYGGAGYTGDDTREKKGIIDQVKDKLPGGQKDDHYSHETHPTTGAYGGAGYTGDDTREKKGVVEK
VKEKLPGGQNVHPTTGPYGGGAAGIGETRERKGVGEKVKEKLPGGHKDDQYLHDTHPTKPTSAAHGGVGHT
GGEPQLHEKKGLIEKIKDKLPGHNN

>Dehydrin-like protein [Prunus persica]; N232
KTDEYGNPVHHTTTGTGRTDEFGNPVQHGVADTGYGTGAGYGIHTKPGVVEHHGVPAVLHSKDDQYSRDTQT
TTGGYGGDGYTGGEHQEKKGLLGQLQDKLPGGNKDGQYSHDTQTTTGAYGGAGYTGGEHHEKKGVIGQVKDK
LPGGQKDDQYCRDTHPTTGAYGGAGYTGGEDHEKKGIIGQVKDKLPGGQKDDQYCRDTHPTTGAYGGAGYTG
GEHQEKKGIIGQVKDKLPGGQKDDQYCHDTHPTTGAYGGAGYTGGDTREKKGIIGQVKDKLPGGQKDDHYSH
DTHPTTGAYGGAGYTGDDTREKKGIIDQVKDKLPGGQKDDHYSHETHPTTGAYGGAGYTGDDTREKKGVVEK
VKEKLPGGQNVHPTTGPYGGGAAGIGETRERKGVGEKVKEKLPGGHKDDQYLHDTHPTKPTSAAHGGVGHT
GGEPQLHEKKGLIEKIKDKLPGHNN

>UTP-glucose-1-phosphate uridylyltransferase [Pyrus pyrifolia]; N246
MAAVATGNVDKLKSDVASLSQISENEKNGFINLVSRYVSGEEAQHVEWSKIQTPTDEVVVPYDGLAPTPEDP
EEIKKLLDKLVVLKLNGGLGTTMGCTGPKSVIEVRNGLTFLDLIVIQIENLNNKYGSCVPLLLMNSFNTHDD
TQKIVEKYSKSNVQIHTFNQSQYPRLVVEDFSPLPSKGQTGKDGWYPPGHGDVFPSLKNSGKLDLLLSQGKE
YVFIANSDNLGAVVDLKILHHLIQKKNEYCMEVTPKTLADVKGGTLISYEGRVQLLEIAQVPDQHVNEFKSI
EKFKIFNTNNLWVNLNAIKRLVEADALKMEIIPNPKEVDGVKVLQLETAAGAAIRFFNHAIGINVPRSRFLP
VKATSDLLLVQSDLYTLQDGFVTRNSARKNPENPTIELGPEFKKVGSYLSRFKSIPSILELESLKVSGDVWF
GAGVVLKGKVTITAKSGVKLEIPDNAVIANKDINGPEDL

Additional file 2 (*continuation*)

>ATPase subunit [Beta vulgaris subsp. vulgaris]; N251
MEFSPRAAELTNLLESRITNFYTNFQVDEIGRVVSVGDGIARVYGLNEIQAGEMVEFASGVKGIALNLENEN
VGIVVFGSDTAIKEGDLVKRTGSIVDVPAGKAMLGRVVDALGVPIDGRGALSDHERRRVEVKAPGIIERKSV
HEPMQTGLKAVDSLVPIGRGQRELIIGDRQTGKTAIAIDTILNQKQLNSKATSESETLYCVYDAVGQKRSTV
AQLVQILSEANALEYSILVAATASDPAPLQFLAPYSGCAMGEYFRDNGMHALIIYDDLSKQAVAYRQMSLLL
RRPPGREAFPGDVFYLHSRLLERAAKRSDQTGAGSLTALPVIETQAGDVSAYIPTNVISITDGQICLETELF
YRGIRPAINVGLSVSRVGSAAQLKAMKQVCGSPKLELAQYREVAAFAQFGSDLDAATQALLNRGARLTEVPK
QPQYAPLPIEKQILVLYGAVNDFCDRMPLDKISQYERTIPNSVKPELLQSLKGGLTNEKKMELDSFLKECAL
NY

>Pyruvate decarboxylase [Fragaria x ananassa]; N255
MDTKIGSIDVCKTENHDVGCLPNSTTSTVQDSVPSTCLSSADATLGRHLARRLVQIGITDVFTVPGDFNLTL
LDHLIAEPGLTNIGCCNELNAGYAADGYARSRGVGACVVTFTVGGLSVLNAIAGAYSENLPVICIVGGPNSN
DYGTNRILHHTIGSPDFSQELRCFQTVTCFQAVVNNLEDAHELIDTAISTALKESKPVYISIGCNLAGIPHP
TFSREPVPFSLSPKLSNKWGLEAAVEAAAEFLNKAVKPVMVGGPKLRSAHAGDAFVKLADASGFALAVMPSA
KGQVPEHHPHFIGTYWGAVSTAFCAEIVESADAYLFAGPIFNDYSSVGYSLLLKKEKAIIVQPDRVTIGNGP
TFGCVLMKDFLVGLAKKLKHNNTAYENYRRIFVPDGHPLKAAPKEPLRVNVLFKHIQKMLSAETAVIAETGD
SWFNCQKLKLPPGCGYEFQMQYGSIGWSVGATLGYAQAVPEKRVIAFIGDGSFQVTAQDVSTMIRNGQRTII
FLINNGGYTIEVEIHDGPYNVIKNWNYTGLVDAIHNGEGKCWTTKVRCEEELIEAIETANGPKKDRLCFIEV
IVHKDDTSKELLEWGSRVSAANSRPPNPQ

>NADP-dependent malic enzyme (NADP-ME) [Vitis vinifera]; N303
MESTLKDIRDGASVLDLDPKATVGGGVEDLYGEDFATEDQLVTPWTVSVASGYSLLRDPRHNKGLAFNDKER
DAHYLCGLLPPVVSTQELQERKLMNSIRQYQVPLQKYMAMMDLQERNERLFYKLLIDNVEELLPVVYTPTVG
EACQKYGSIFRRPQGLYISLKEKGKILEVLKNWPERRIQVIVVTDGERILGLGDLGCQGMGIPVGKLSLYTA
LGGVRPSACLPITIDVGTNNEKLLANEFYIGLKQRRATGKEYSEFLQEFMSPVKQNYGEKVLIQFEDFANHN
AFDLLAKYGTTHLAFNDDIQGTASVVLAGIVSALRLLGGTLADHKFLFLGAGEAGTGIAELIALEMSKQTKC
PIEETRKKIWLVDSKGLIVGSRKDSLQQFKKPWAHEHEPVKDLLDAVKVIKPTVLIGSSGVGKAFTKEVIEA
MASCNEKPLILALSNPTSQSECTAEEAYTWTQGRAIFASGSPFDPVEYNGKTFVPGQANNAYIFPGLGMGLV
ISGAIRVHDEMLLAASEALARQVTQENFDKGLIYPPFSNIRKISAHIAANVAAKAYELGLATRLPQPENLVK
YAESCMYSPVYRSYR

>NADP-dependent malic enzyme (NADP-ME) [Vitis vinifera]; N304
MESTLKDIRDGASVLDLDPKATVGGGVEDLYGEDFATEDQLVTPWTVSVASGYSLLRDPRHNKGLAFNDKER
DAHYLCGLLPPVVSTQELQERKLMNSIRQYQVPLQKYMAMMDLQERNERLFYKLLIDNVEELLPVVYTPTVG
EACQKYGSIFRRPQGLYISLKEKGKILEVLKNWPERRIQVIVVTDGERILGLGDLGCQGMGIPVGKLSLYTA
LGGVRPSACLPITIDVGTNNEKLLANEFYIGLKQRRATGKEYSEFLQEFMSPVKQNYGEKVLIQFEDFANHN
AFDLLAKYGTTHLAFNDDIQGTASVVLAGIVSALRLLGGTLADHKFLFLGAGEAGTGIAELIALEMSKQTKC
PIEETRKKIWLVDSKGLIVGSRKDSLQQFKKPWAHEHEPVKDLLDAVKVIKPTVLIGSSGVGKAFTKEVIEA
MASCNEKPLILALSNPTSQSECTAEEAYTWTQGRAIFASGSPFDPVEYNGKTFVPGQANNAYIFPGLGMGLV
ISGAIRVHDEMLLAASEALARQVTQENFDKGLIYPPFSNIRKISAHIAANVAAKAYELGLATRLPQPENLVK
YAESCMYSPVYRSYR

>Pyruvate decarboxylase [Fragaria x ananassa]; N305
MDTKIGSIDVCKTENHDVGCLPNSTTSTVQDSVPSTCLSSADATLGRHLARRLVQIGITDVFTVPGDFNLTL
LDHLIAEPGLTNIGCCNELNAGYAADGYARSRGVGACVVTFTVGGLSVLNAIAGAYSENLPVICIVGGPNSN
DYGTNRILHHTIGSPDFSQELRCFQTVTCFQAVVNNLEDAHELIDTAISTALKESKPVYISIGCNLAGIPHP
TFSREPVPFSLSPKLSNKWGLEAAVEAAAEFLNKAVKPVMVGGPKLRSAHAGDAFVKLADASGFALAVMPSA
KGQVPEHHPHFIGTYWGAVSTAFCAEIVESADAYLFAGPIFNDYSSVGYSLLLKKEKAIIVQPDRVTIGNGP
TFGCVLMKDFLVGLAKKLKHNNTAYENYRRIFVPDGHPLKAAPKEPLRVNVLFKHIQKMLSAETAVIAETGD
SWFNCQKLKLPPGCGYEFQMQYGSIGWSVGATLGYAQAVPEKRVIAFIGDGSFQVTAQDVSTMIRNGQRTII
FLINNGGYTIEVEIHDGPYNVIKNWNYTGLVDAIHNGEGKCWTTKVRCEEELIEAIETANGPKKDRLCFIEV
IVHKDDTSKELLEWGSRVSAANSRPPNPQ

Additional file 2 (*continuation*)

>Thaumatin-like protein [Prunus persica]; N310
MMKSQAALLGLTTLAILFFSGAHAAKITFTNKCSYTVWPGTLTGDQKPQLSLTGFELATGISRSVDAPSPWS
GRFFGRTRCSTDASGKFTCATADCGSGQVSCNGNGAAPPATLVEITIASNGGQDFYDVSLVDGFNLPMSVAP
QGGTGKCKASTCPADINKVCPAPLQVKGSDGSVIACKSACLAFNQPKYCCTPPNDKPETCPPPDYSKLFKTQ
CPQAYSYAYDDKSSTFTCSGRPAYLITFCP

>Glutathione S-transferase [Cucurbita maxima]; N313
MADEVKLLDFWPSMFGIRVRIALAEKGVRIEYMEQDLRNKSPLLLQMNPVHKKIPVLVHNGRPICESSIIVQ
YIDEVWKDKAPLLPSDPYQRAQARFWVDFIDKKLYDAGRKVWTGKGEEVEAGKELIGVLKQLEEVLGEKGFF
GGERLGFVDIALIGFHSWFYTYEAFGKLSIEAECPKIMGWAKRCLEKESVSKSLPDSKKVYDFAVQMKKALG
LE

>Putative glycine-rich RNA binding protein 1 [Catharanthus roseus]; N322
MASADVEFRCFVGGLAWATTDQSLSEAFSQYGEVLESKIINDRETGRSRGFGFVTFGDEKSMKDAIEGMNGQ
TLDGRNVTVNEAQSRGSGGGGGGGGFRGPRREGGGCYGGGGRRNGGYGGNGGGYGGGRRDGGRRW

>Hypothetical protein [Oryza sativa (japonica cultivar-group)]; N323
MPATRASRCTSDPCHPGWIWTAGVCRRWLATVAADNGGWTVVAGWGCPWRWRWRWRWWLTVVAGDGGGCG
SGGGGCRRWYLGRRWLKAIIRLTVRHRRRRYVEAGKGSRGGEGGVWVRARRRRREQLWAGIAGESLAEPF
GQLMASFPSLEALF
