# Supplementary material for: Proteomic analysis of peach fruit mesocarp softening and chilling injury using difference gel electrophoresis (DIGE)
Source: BMC Genomics. 2010 Jan 18;11:43. doi: 10.1186/1471-2164-11-43 (PMC2822761; doi:10.1186/1471-2164-11-43)
Supplement: Additional file 3 — Differential accumulation of each spot detected in the 2-D gels analysis from peach fruit mesocarp. The experimental values of isolelectrical point (pI) and molecular weight (MW) of all the spots analyzed, as well as its accumulation pattern and the PCA weight loadings values are listed. [file 1471-2164-11-43-S3.PDF]

Additional file 3

Label	pI	MW	E1 v E2	E1 v E3	E1 v E4	E2 v E3	E2 v E4	E3 v E4	PC1	PC2	PC3
N001	8.3	19.4	0	0	0	0	0	0	0.08	-0.04	-0.06
N002	8.3	19.4	0	0	0	0	0	0	0.14	-0.06	-0.21
N003	8.2	19.5	0	0	0	0	0	0	0.08	-0.02	-0.03
N004	8.1	20.0	**-1**	**1**	0	**1**	**1**	**-1**	-0.06	-0.08	0.08
N005	7.9	21.1	**-1**	**-1**	**-1**	**1**	**1**	0	-0.05	-0.05	-0.04
N006	7.8	19.8	0	0	0	0	0	0	-0.06	-0.02	0.03
N007	7.9	17.2	0	0	0	0	0	0	-0.02	0.07	-0.03
N008	7.8	17.8	0	0	0	0	0	0	-0.14	0.07	-0.09
N009	7.2	16.3	0	0	0	0	0	0	0.07	0.05	-0.01
N011	7.3	21.2	**-1**	0	**-1**	**1**	**1**	**-1**	-0.12	-0.10	0.06
N012	7.2	22.0	**1**	**-1**	**-1**	**-1**	**-1**	0	0.00	0.07	-0.05
N013	7.3	22.2	**1**	0	**1**	**-1**	0	**1**	0.08	0.01	-0.02
N014	7.1	19.6	0	0	0	0	0	0	0.02	-0.06	0.07
N016	6.9	18.6	0	0	0	0	0	0	0.10	-0.08	0.17
N017	6.9	18.9	0	0	0	0	0	0	0.05	0.04	0.02
N018	7.9	24.6	0	0	0	0	0	0	0.05	0.01	0.02
N019	6.8	19.7	0	0	0	0	0	0	0.07	-0.04	-0.02
N020	6.6	19.2	0	0	0	0	0	0	0.03	0.00	0.04
N021	6.6	18.4	0	0	0	0	0	0	0.00	-0.07	0.06
N022	6.7	20.2	**-1**	0	**-1**	0	0	**-1**	-0.10	-0.08	0.05
N023	6.7	20.9	**-1**	0	**-1**	**1**	**1**	**-1**	-0.11	-0.10	0.05
N024	6.8	23.0	0	0	0	0	0	0	-0.01	0.14	0.01
N025	6.9	22.0	0	0	0	0	0	0	0.02	0.09	0.04
N026	6.5	20.4	0	0	0	0	0	0	0.07	0.02	0.03
N027	6.3	22.0	**-1**	**-1**	**-1**	**1**	**1**	0	-0.07	-0.06	-0.04
N028	6.3	19.7	0	0	0	0	0	0	0.01	0.00	0.17
N029	6.4	19.0	**1**	**1**	**1**	0	0	0	0.06	-0.02	0.10
N030	6.4	18.5	0	0	0	0	0	0	0.07	-0.02	0.10
N031	6.3	19.0	**-1**	**-1**	**-1**	**1**	**1**	0	-0.13	-0.17	-0.03
N032	6.3	18.5	**-1**	**-1**	**-1**	**1**	**1**	0	-0.09	-0.10	-0.02
N033	6.4	17.0	0	0	0	0	0	0	0.06	0.00	-0.04
N034	6.1	18.5	0	0	0	0	0	0	0.06	-0.04	-0.04
N035	6.0	18.1	**-1**	**-1**	**-1**	**1**	**1**	0	-0.07	-0.08	-0.02
N036	6.0	17.4	**-1**	**-1**	**-1**	**1**	**1**	**-1**	-0.06	-0.03	0.00
N037	6.0	20.0	**-1**	0	0	**1**	**1**	0	-0.07	-0.09	0.03
N039	5.6	18.3	0	0	0	0	0	0	-0.14	0.00	-0.12
N041	5.6	17.5	0	0	0	0	0	0	0.06	0.02	-0.02
N042	7.7	28.0	**-1**	0	**-1**	**1**	**1**	**-1**	-0.08	-0.07	0.04
N044	8.3	28.7	0	0	0	0	0	0	0.07	0.03	0.06
N046	8.1	30.0	0	0	0	0	0	0	-0.04	-0.03	0.06
N047	7.9	29.5	0	0	0	0	0	0	-0.01	-0.01	-0.02
N048	7.6	31.9	0	0	0	0	0	0	0.04	0.03	-0.20
N049	7.3	29.5	0	0	0	0	0	0	0.08	0.01	0.00
N051	7.2	28.1	0	0	0	0	0	0	0.06	-0.03	-0.01
N052	7.1	29.4	0	0	0	0	0	0	0.08	0.01	0.04
N053	6.9	27.4	0	0	0	0	0	0	0.09	-0.04	0.07
N054	6.7	27.2	0	0	0	0	0	0	0.12	0.03	0.06
N055	6.6	27.9	0	0	0	0	0	0	-0.05	0.08	0.00
N056	6.6	26.8	0	0	0	0	0	0	0.05	0.03	-0.07
N057	6.4	25.7	0	0	0	0	0	0	0.05	0.02	-0.11
N058	5.7	20.5	0	0	0	0	0	0	0.03	0.02	0.02
N059	5.5	23.8	0	0	0	0	0	0	0.09	-0.06	0.08
N060	5.3	22.3	0	0	0	0	0	0	-0.01	0.00	-0.04
N061	7.2	23.0	0	0	0	0	0	0	-0.01	0.08	-0.02
N062	5.8	18.4	0	0	0	0	0	0	-0.04	0.07	0.01
N063	6.1	24.3	0	0	0	0	0	0	0.11	0.05	0.02
N064	8.1	32.7	0	0	0	0	0	0	0.05	0.00	-0.08
N065	8.2	32.4	0	0	0	0	0	0	0.03	0.01	-0.05
N066	8.3	32.4	0	0	0	0	0	0	0.00	-0.05	0.03
N067	8.4	33.2	0	0	0	0	0	0	-0.06	0.10	0.04
N068	8.3	35.0	0	0	0	0	0	0	0.00	-0.03	0.02
N069	8.2	34.3	0	0	0	0	0	0	-0.01	0.09	0.00
N070	8.1	34.3	0	0	0	0	0	0	0.03	0.08	0.08
N071	8.1	37.3	0	0	0	0	0	0	0.07	0.06	-0.04
N072	8.1	37.2	0	0	0	0	0	0	0.03	0.03	-0.10
N073	8.1	36.3	**-1**	**-1**	0	**-1**	**1**	**1**	0.00	-0.04	-0.13

Additional file 3 (*continuation*)

Label	pI	MW	E1 v E2	E1 v E3	E1 v E4	E2 v E3	E2 v E4	E3 v E4	PC1	PC2	PC3
N074	7.9	34.0	0	0	0	0	0	0	-0.02	0.06	-0.08
N076	7.8	33.8	0	0	0	0	0	0	0.06	0.08	0.06
N077	7.4	34.2	**1**	0	**1**	**-1**	0	**1**	0.06	-0.01	-0.03
N078	7.2	31.8	0	0	0	0	0	0	0.04	-0.06	-0.05
N079	7.8	32.6	0	0	0	0	0	0	0.05	-0.04	-0.04
N080	7.1	33.1	0	0	0	0	0	0	0.05	0.09	-0.01
N081	7.1	32.3	0	0	0	0	0	0	-0.01	0.03	0.02
N082	7.0	31.2	0	0	0	0	0	0	0.04	0.04	0.07
N083	6.9	30.6	0	0	0	0	0	0	-0.01	-0.05	0.00
N084	6.8	31.6	0	0	0	0	0	0	0.08	-0.01	0.04
N086	6.5	30.2	0	0	0	0	0	0	0.02	0.01	0.13
N087	6.4	29.0	0	0	0	0	0	0	0.08	-0.04	0.01
N089	6.3	29.0	0	0	0	0	0	0	0.01	0.02	0.17
N093	6.2	31.1	0	0	**1**	**-1**	**1**	**1**	0.07	-0.06	-0.07
N094	6.2	30.3	0	0	0	0	0	0	0.05	0.00	-0.02
N095	6.2	29.8	**1**	0	**1**	**-1**	**1**	**1**	0.06	-0.02	-0.04
N096	6.3	27.4	0	0	0	0	0	0	-0.02	0.03	-0.10
N097	6.1	30.4	0	0	0	0	0	0	0.05	0.00	-0.01
N098	5.9	30.3	0	0	0	0	0	0	0.08	0.02	0.02
N100	5.8	29.9	0	0	0	0	0	0	0.06	0.04	0.01
N101	6.0	28.1	0	0	0	0	0	0	0.04	0.04	0.04
N102	6.0	27.2	0	0	0	0	0	0	0.06	0.03	-0.05
N103	5.9	26.6	0	0	0	0	0	0	-0.03	0.07	0.03
N104	5.7	26.5	0	0	0	0	0	0	-0.10	0.06	-0.01
N106	5.8	33.5	0	0	0	0	0	0	0.03	0.06	-0.01
N107	5.6	33.3	0	0	0	0	0	0	0.04	0.07	-0.03
N110	5.9	33.5	0	0	0	0	0	0	-0.01	0.11	-0.01
N111	5.8	29.3	**1**	**1**	**1**	**-1**	**-1**	**1**	0.06	0.02	-0.01
N112	5.8	36.1	0	0	0	0	0	0	0.05	-0.04	-0.01
N113	5.6	37.4	0	0	0	0	0	0	-0.02	-0.02	-0.01
N114	5.5	39.0	0	0	0	0	0	0	-0.03	0.06	-0.09
N115	5.5	37.9	0	0	0	0	0	0	0.02	-0.02	-0.16
N116	5.5	36.5	0	0	0	0	0	0	0.05	0.01	-0.04
N117	5.3	39.8	0	0	0	0	0	0	0.04	-0.01	0.04
N118	5.3	37.2	0	0	0	0	0	0	-0.05	0.01	0.06
N119	5.3	35.1	0	0	0	0	0	0	-0.02	0.06	-0.05
N120	5.3	33.5	0	0	0	0	0	0	-0.02	0.06	-0.02
N121	5.2	36.8	0	0	0	0	0	0	0.01	0.01	-0.15
N122	5.2	31.6	0	0	0	0	0	0	-0.06	0.06	0.00
N124	6.5	33.9	0	0	0	0	0	0	-0.04	0.12	0.01
N125	6.7	33.1	0	0	0	0	0	0	0.03	0.03	0.07
N127	5.8	38.0	0	0	0	0	0	0	-0.07	-0.01	0.03
N128	6.0	39.7	0	0	0	0	0	0	-0.12	0.01	-0.05
N129	5.9	37.6	0	0	0	0	0	0	-0.06	-0.03	-0.02
N131	6.1	37.6	0	0	0	0	0	0	0.02	0.05	0.00
N132	6.5	41.8	0	0	0	0	0	0	0.01	0.05	0.07
N133	6.5	41.0	0	0	0	0	0	0	0.00	0.05	0.08
N134	6.6	36.9	0	0	0	0	0	0	-0.02	0.07	-0.02
N135	6.6	39.9	0	0	0	0	0	0	0.05	-0.01	0.00
N136	6.7	41.5	0	0	0	0	0	0	0.01	0.04	0.04
N137	6.8	39.4	0	0	0	0	0	0	0.02	0.07	0.04
N138	6.7	38.7	**1**	**1**	**1**	0	**-1**	0	0.09	0.05	0.08
N139	6.9	38.8	0	0	0	0	0	0	0.10	-0.03	0.00
N140	7.0	39.5	0	0	0	0	0	0	-0.01	0.05	0.13
N141	7.0	37.0	0	0	0	0	0	0	-0.10	0.07	0.09
N142	7.0	35.9	0	0	0	0	0	0	-0.05	0.03	0.05
N143	7.0	35.6	0	0	0	0	0	0	-0.05	0.12	0.03
N144	6.9	36.8	0	0	0	0	0	0	0.05	0.01	0.08
N146	7.3	39.8	0	0	0	0	0	0	-0.09	-0.05	-0.06
N147	7.2	40.1	0	0	0	0	0	0	0.05	-0.01	-0.04
N148	7.2	39.6	0	0	0	0	0	0	0.00	-0.08	-0.01
N149	7.5	39.5	0	0	0	0	0	0	-0.02	-0.04	0.03
N150	7.5	38.9	0	0	0	0	0	0	-0.01	-0.01	0.16
N151	7.5	37.2	0	0	0	0	0	0	-0.09	0.14	0.20
N152	7.9	39.5	0	0	0	0	0	0	0.01	0.07	-0.05
N153	7.6	41.1	0	0	0	0	0	0	0.06	0.05	0.00

Additional file 3 (*continuation*)

Label	pI	MW	E1 v E2	E1 v E3	E1 v E4	E2 v E3	E2 v E4	E3 v E4	PC1	PC2	PC3
N154	7.7	42.4	0	0	0	0	0	0	-0.11	0.02	0.02
N155	7.6	42.2	0	0	0	0	0	0	0.00	0.05	0.02
N156	7.5	42.3	0	0	0	0	0	0	0.01	0.05	0.07
N157	7.2	41.2	0	0	0	0	0	0	0.05	-0.02	0.00
N158	7.2	42.2	0	0	0	0	0	0	0.06	0.02	-0.03
N162	5.5	43.9	0	**-1**	**-1**	**1**	0	**-1**	-0.05	0.00	0.07
N163	5.6	43.7	**-1**	0	**-1**	**1**	**1**	**-1**	-0.07	-0.04	0.05
N164	5.7	43.7	**-1**	0	**-1**	**1**	**1**	**-1**	-0.06	-0.04	0.04
N165	5.9	42.6	0	**-1**	**-1**	**-1**	**-1**	0	-0.04	0.08	-0.05
N166	5.6	46.3	0	0	0	0	0	0	0.03	0.09	0.05
N167	5.7	46.3	0	0	0	0	0	0	0.03	0.12	0.09
N168	5.6	48.4	0	0	0	0	0	0	0.06	0.03	0.05
N169	5.7	49.3	0	0	0	0	0	0	0.06	0.02	0.03
N170	5.8	49.2	0	0	0	0	0	0	0.03	-0.03	-0.05
N172	6.0	48.7	0	0	0	0	0	0	0.03	-0.02	-0.13
N173	6.2	48.4	0	0	0	0	0	0	0.04	0.06	0.06
N174	6.2	49.1	0	0	0	0	0	0	0.02	0.10	-0.07
N176	6.3	49.1	0	0	0	0	0	0	0.03	0.08	-0.06
N177	6.3	48.3	0	0	0	0	0	0	-0.05	0.03	0.04
N178	6.4	49.1	0	0	0	0	0	0	0.02	0.04	-0.05
N181	6.4	45.5	0	0	0	0	0	0	-0.05	-0.04	0.00
N182	6.3	44.8	0	0	0	0	0	0	0.12	0.03	0.04
N183	6.2	45.4	0	0	**-1**	0	**-1**	**-1**	-0.09	0.10	0.09
N184	6.1	44.8	0	0	0	0	0	0	0.06	0.02	0.07
N185	6.0	45.5	0	0	0	0	0	0	0.04	0.02	0.05
N186	6.5	45.6	0	0	0	0	0	0	0.03	0.00	0.02
N187	6.1	47.2	0	0	**-1**	0	**-1**	**-1**	-0.08	0.10	-0.01
N190	6.7	49.7	0	0	0	0	0	0	-0.01	0.00	-0.07
N192	6.9	49.5	0	0	0	0	0	0	-0.07	-0.12	0.10
N193	6.9	48.9	**-1**	0	**-1**	**1**	**1**	**-1**	-0.24	-0.36	0.11
N199	6.8	45.9	0	0	0	0	0	0	0.01	0.03	-0.02
N200	7.0	50.2	0	0	0	0	0	0	-0.02	-0.04	0.04
N201	7.0	49.1	**-1**	0	0	**1**	**1**	0	-0.11	-0.17	-0.01
N202	7.1	46.1	0	0	0	0	0	0	-0.01	-0.07	0.01
N204	7.2	50.2	0	0	0	0	0	0	0.11	0.01	0.09
N205	7.2	50.5	0	0	0	0	0	0	-0.05	0.02	0.04
N206	7.3	50.1	0	0	0	0	0	0	0.00	-0.01	-0.09
N207	7.4	50.1	0	0	0	0	0	0	0.11	0.03	0.19
N208	7.2	47.9	0	**-1**	**-1**	**-1**	**-1**	0	-0.01	0.07	-0.06
N209	7.7	47.6	0	0	**-1**	0	**-1**	**-1**	-0.10	0.19	0.01
N210	7.6	48.1	0	0	0	0	0	0	-0.08	0.04	0.01
N211	7.6	48.2	0	0	0	0	0	0	-0.06	0.03	0.04
N213	7.9	50.4	0	0	0	0	0	0	-0.09	0.02	-0.04
N214	7.8	50.3	0	0	0	0	0	0	-0.05	-0.02	0.00
N217	8.1	49.4	0	0	0	0	0	0	-0.05	0.01	-0.03
N222	8.2	46.1	**1**	**1**	**1**	0	0	0	0.08	-0.01	0.05
N223	8.3	46.0	0	0	0	0	0	0	0.01	-0.06	-0.08
N224	7.6	44.0	0	0	0	0	0	0	-0.01	0.09	0.03
N225	7.4	43.9	0	0	0	0	0	0	0.00	0.04	0.07
N228	7.6	57.9	**1**	**1**	**1**	0	**1**	**1**	0.10	-0.06	0.03
N229	7.6	58.0	**1**	**1**	**1**	0	**1**	**1**	0.08	-0.03	0.03
N230	7.5	58.1	0	0	0	0	0	0	0.09	0.01	0.03
N231	7.4	61.7	**-1**	**-1**	**-1**	**-1**	**-1**	**1**	-0.02	0.04	-0.07
N232	7.4	61.7	0	**-1**	**-1**	**-1**	**-1**	0	-0.02	0.05	-0.09
N233	7.0	55.0	0	0	0	0	0	0	-0.06	0.01	0.00
N234	7.0	56.3	0	0	0	0	0	0	-0.02	0.06	-0.06
N235	7.6	55.2	0	0	0	0	0	0	-0.01	0.06	0.05
N236	7.7	55.2	0	0	0	0	0	0	-0.16	0.19	-0.09
N237	6.9	59.9	0	0	0	0	0	0	0.07	-0.01	0.02
N238	6.8	60.7	0	0	0	0	0	0	0.06	0.08	0.01
N239	6.7	60.1	0	0	0	0	0	0	0.02	0.06	0.04
N240	6.7	59.1	0	0	0	0	0	0	0.01	0.03	-0.06
N241	6.7	57.7	0	0	0	0	0	0	0.00	0.07	-0.01
N242	6.8	56.5	0	0	0	0	0	0	0.07	0.01	0.15
N243	6.6	57.3	0	0	0	0	0	0	-0.01	0.03	0.01

Additional file 3 (*continuation*)

Label	pI	MW	E1 v E2	E1 v E3	E1 v E4	E2 v E3	E2 v E4	E3 v E4	PC1	PC2	PC3
N244	6.5	57.4	0	0	0	0	0	0	0.05	0.01	-0.05
N246	6.5	55.7	0	0	0	0	0	0	0.03	0.01	0.01
N251	6.5	54.8	0	0	0	0	0	0	0.08	0.02	0.00
N253	7.5	66.5	0	0	0	0	0	0	-0.05	0.06	-0.03
N255	6.9	71.3	**-1**	**-1**	**-1**	**1**	**-1**	**-1**	-0.09	0.05	0.06
N256	7.0	69.9	0	0	0	0	0	0	0.07	-0.08	-0.06
N259	5.7	51.6	0	0	0	0	0	0	0.05	0.04	0.02
N260	5.8	51.2	0	0	0	0	0	0	0.06	0.06	-0.07
N261	5.9	51.2	0	0	0	0	0	0	-0.01	0.07	-0.10
N264	6.2	51.2	0	0	0	0	0	0	-0.01	0.06	-0.06
N266	6.1	56.5	0	0	0	0	0	0	-0.10	0.02	0.02
N268	6.0	56.5	0	0	0	0	0	0	0.05	0.05	0.09
N269	6.0	59.7	0	0	0	0	0	0	-0.07	0.03	0.02
N270	6.0	57.3	0	0	0	0	0	0	0.02	0.08	0.05
N273	6.0	53.0	0	0	0	0	0	0	-0.06	0.07	-0.03
N275	5.5	55.7	0	0	0	0	0	0	0.17	-0.01	0.09
N277	5.6	58.9	0	0	0	0	0	0	0.02	0.05	-0.07
N278	5.7	59.1	0	0	0	0	0	0	0.04	0.03	-0.11
N280	5.8	56.5	0	0	0	0	0	0	0.10	-0.04	0.00
N281	5.9	56.6	0	0	0	0	0	0	0.08	0.02	-0.05
N282	5.9	59.8	0	0	0	0	0	0	0.10	0.05	-0.03
N283	5.8	59.7	0	0	0	0	0	0	-0.03	0.12	0.03
N285	5.6	67.4	0	0	0	0	0	0	0.00	0.03	-0.10
N287	5.6	69.3	0	0	0	0	0	0	-0.08	0.09	0.06
N288	5.5	67.4	0	0	0	0	0	0	-0.05	0.08	0.06
N289	5.3	67.0	0	0	0	0	0	0	0.03	-0.01	-0.02
N290	5.3	68.8	0	0	0	0	0	0	0.02	-0.01	-0.03
N291	5.4	72.0	0	0	0	0	0	0	0.04	0.02	-0.05
N292	5.2	69.0	0	0	0	0	0	0	-0.06	0.05	0.02
N293	5.4	63.4	0	0	0	0	0	0	-0.08	0.14	0.00
N295	5.7	62.2	0	0	0	0	0	0	-0.04	0.04	0.04
N296	5.9	65.3	0	0	0	0	0	0	0.03	-0.05	-0.07
N297	5.8	65.7	0	0	0	0	0	0	-0.02	0.07	-0.01
N299	7.1	34.2	0	0	0	0	0	0	-0.01	0.03	-0.01
N300	7.0	25.6	0	0	0	0	0	0	-0.06	-0.02	0.06
N301	5.2	28.1	0	0	0	0	0	0	-0.05	-0.01	0.06
N302	7.2	54.7	0	0	0	0	0	0	-0.02	0.04	0.01
N303	6.4	65.4	0	0	0	0	0	0	0.06	-0.01	-0.02
N304	6.3	65.6	**1**	**1**	**1**	**-1**	0	**1**	0.08	0.00	0.02
N305	6.3	61.8	**-1**	0	**-1**	**1**	**-1**	**-1**	-0.06	0.02	0.02
N310	7.0	33.2	0	0	**-1**	0	**-1**	**-1**	-0.21	0.26	0.04
N313	6.1	28.3	**1**	**1**	**1**	**-1**	**-1**	0	0.06	0.06	0.05
N322	6.1	16.4	**-1**	**-1**	**-1**	**-1**	**-1**	0	-0.03	0.04	-0.07
N323	6.1	15.7	**-1**	**-1**	**-1**	**-1**	**-1**	0	-0.03	0.05	-0.07
N324	7.3	71.8	0	0	0	0	0	0	0.05	0.03	0.08

		E1 v E2	E1 v E3	E1 v E4	E2 v E3	E2 v E4	E3 v E4
**Increase**	**-1**	**22**	**16**	**27**	**15**	**16**	**16**
**No change**	**0**	**207**	**216**	**202**	**210**	**205**	**215**
**Decrease**	**1**	**12**	**9**	**12**	**18**	**20**	**10**

PC1, PC2 and PC3 columns displays the loading values (weight) for each spot for the first, second and third PCA components, respectively.
