# Supplementary material for: Proteomic analysis of peach fruit mesocarp softening and chilling injury using difference gel electrophoresis (DIGE)
Source: BMC Genomics. 2010 Jan 18;11:43. doi: 10.1186/1471-2164-11-43 (PMC2822761; doi:10.1186/1471-2164-11-43)
Supplement: Additional file 4 — Gene Ontology Annotation. A detailed description of each biological process assigned to the differentially accumulated proteins is listed together with the evidence source used by the GO annotation system. [file 1471-2164-11-43-S4.PDF]

Additional file 4

Spot No.	Evidence	GO Name
**N004, N011, N027, N037**	IEA	response to stress
**N005**	IEA	metabolic process
	IEA	carbohydrate metabolic process
	IEA	carbohydrate metabolic process
	IEA	metabolic process
**N012**	IEA	signal transduction
**N023**	IEA	response to stress
**N029**	IEA	response to stress
	IEA	response to biotic stimulus
	IEA	response to stress
	IEA	response to biotic stimulus
**N031**	IEA	response to stress
**N035**	IEA	cellular homeostasis
	IEA	cellular homeostasis
	IEA	cellular homeostasis
	IEA	cellular homeostasis
	IEA	cellular homeostasis
	IEA	cellular homeostasis
	IEA	metabolic process
**N042**	IEA	metabolic process
	IEA	generation of precursor metabolites and energy
	IEA	generation of precursor metabolites and energy
	IEA	generation of precursor metabolites and energy
	IEA	metabolic process
**N073**	IEA	cellular process
	IEA	cellular component organization
**N077**	IEA	transport
**N093**	IEA	response to stress
**N111**	IEA	cellular homeostasis
	IEA	transport
	IEA	cellular homeostasis
	IEA	cellular homeostasis
**N162, N163 and N164**	IEA	metabolic process
	IEA	metabolic process
	IEA	metabolic process
	IEA	metabolic process
	IEA	metabolic process
	IEA	cellular amino acid and derivative metabolic process
	IEA	cellular amino acid and derivative metabolic process
	IEA	cellular amino acid and derivative metabolic process
	IEA	metabolic process
	IEP	biological_process
	IEP	response to stress
	IEP	response to abiotic stimulus
	IEP	response to abiotic stimulus
	IMP	metabolic process
**N165**	IEA	metabolic process
	IEP	response to stress
	IEP	response to abiotic stimulus
	IEA	metabolic process
	IEA	metabolic process

Additional file 4 (*continuation*)

Spot No.	Evidence	GO Name
**N183**	IEA	cellular process
	IEA	cellular component organization
	IEA	carbohydrate metabolic process
	IEA	biosynthetic process
	IEA	cellular process
	IEA	cellular process
	IEA	cellular component organization
	IEA	carbohydrate metabolic process
	IEA	biosynthetic process
	IEA	cellular process
**N187**	IEA	metabolic process
**N193**	IEA	metabolic process
	IEA	cellular process
	IEA	cellular component organization
	IEA	carbohydrate metabolic process
**N201**	IEA	metabolic process
	IEA	metabolic process
	IEA	metabolic process
	IEA	metabolic process
	IEA	metabolic process
	IEA	cellular process
**N208**	IEA	metabolic process
**N209**	IEA	cellular amino acid and derivative metabolic process
	IEA	biosynthetic process
	IEA	metabolic process
	IEA	cellular amino acid and derivative metabolic process
	IEA	biosynthetic process
	IEA	cellular amino acid and derivative metabolic process
	IEA	biosynthetic process
**N222**	IEA	metabolic process
	IEA	metabolic process
**N228 and N229**	IEA	response to stress
	IEA	catabolic process
	IEA	cellular process
	IEA	metabolic process
	IEA	response to stress
	IEA	response to stress
	IEA	response to stress
**N231 and N232**	IEA	response to stress
	IEA	response to abiotic stimulus
**N304**	IEA	metabolic process
	IEA	cellular process
	IEA	metabolic process
	IEA	metabolic process
	IEA	metabolic process
**N310**	IEP	response to stress
	IEA	response to stress

IEA: Inferred from Electronic Annotation
IEP: Inferred from Expression Pattern
IMP: Inferred from Mutant Phenotype
